# A neuregulin-like ligand and EGF receptor underpin *Echinococcus multilocularis* development

**DOI:** 10.3389/fcimb.2026.1742233

**Published:** 2026-02-20

**Authors:** Akito Koike, Monika Bergmann, Katia Cailliau, Jérôme Vicogne, Frank Becker, Colette Dissous, Stefan Hannus, Klaus Brehm

**Affiliations:** 1 Consultant Laboratory for Echinococcosis, Institute of Hygiene and Microbiology, University of Würzburg, Würzburg, Germany; 2 Univ. Lille, CNRS, UMR 8576-UGSF-Unité de Glycobiologie Structurale et Fonctionnelle, Lille, France; 3 University of Lille, CNRS, Inserm, CHU Lille, Institut Pasteur de Lille, U1019-UMR 9017, Center for Infection and Immunity of Lille, Lille, France; 4 Intana Bioscience GmbH, Martinsried, Germany

**Keywords:** afatinib, echinococcosis, *Echinococcus* EGF signaling, epidermal growth factor signaling, neuregulin, stem cell

## Abstract

Alveolar echinococcosis (AE), a neglected zoonotic disease, is caused by the infiltrative growth of the metacestode larval stage of the cestode *Echinococcus multilocularis* within host organs. We previously showed that metacestode development relies on the mitotic activity of a population of parasite stem cells known as germinative cells. However, the molecular mechanisms that control *Echinococcus* stem cell dynamics such as cell-cycle progression, self-renewal, and differentiation remain poorly understood. Building on earlier reports implicating epidermal growth factor (EGF) signalling in stem cell regulation, we here characterize the parasite’s repertoire of three EGF receptors. Using RNAi and inhibitor assays, we identify one receptor, EmER1, as essential for the formation of metacestode vesicles from germinative cells. We further demonstrate that EmER1 is targeted by afatinib, an EGF receptor inhibitor with potent anti-parasitic activity both *in vitro* and *in vivo*. Through bioinformatic analyses and membrane-bound yeast two-hybrid assays, we identified a parasite-derived, neuregulin-like ligand for EmER1, termed EmNRG, whose expression is strongly upregulated in metacestode vesicles during clonal expansion of germinative cells. RNAi-mediated knockdown of the EmNRG encoding gene severely impaired the capacity of germinative cells to generate metacestode vesicles. Our data support that EmNRG and EmER1 constitute a cognate ligand - receptor system regulating the balance between asymmetric and symmetric stem cell division in *E. multilocularis*. These findings provide new insights into *Echinococcus* stem cell biology and highlight EGF signalling as a promising avenue for developing novel anti-echinococcosis therapeutics.

## Introduction

Alveolar echinococcosis (AE) is a potentially lethal zoonosis caused by the larval stage of the fox tapeworm *Echinococcus multilocularis*. Infection of intermediate hosts (rodents and humans) begins with ingestion of parasite eggs containing the oncosphere stage (Thompson, 2017). After hatching in the intestine, the oncosphere penetrates the intestinal barrier and disseminates to internal organs, most often the liver, where it transforms into the metacestode stage (Brehm and Koziol, 2017; Thompson, 2017). The metacestode is essentially a cyst-like structure, consisting of an acellular laminated layer that provides host protection and an inner cellular germinal layer composed of a limited number of cell types, including stem, muscle, nerve, glycogen-storing cells, and a syncytial tegument (Brehm and Koziol, 2017). In *E. multilocularis*, the multivesicular metacestode tissue proliferates in a cancer-like, infiltrative manner within the liver. We previously demonstrated that this proliferation is driven exclusively by a stem cell population, termed germinative cells, which are the sole mitotically active cells of the parasite and give rise to all differentiated lineages (Koziol et al., 2014). This study, together with more recent transcriptomic analyses of germinative cells (Herz et al., 2024), revealed that the *Echinococcus* stem cell system shares many features, though also striking differences, with the neoblasts of free-living flatworms. Germinative cells are thought to be relatively resistant to the benzimidazoles albendazole and mebendazole, the current standard therapies for AE. Consequently, novel treatments must primarily target the parasite’s stem cell system to achieve parasitocidal efficacy (Brehm and Koziol, 2014; Koziol and Brehm, 2015). Because of their central role in parasite persistence, the intrinsic mechanisms governing germinative cell renewal and differentiation into muscle, nerve, and tegumental lineages are of considerable medical and biological interest.

Among the evolutionarily conserved signalling pathways regulating stem cell fate in metazoans is the epidermal growth factor (EGF) pathway. This involves secreted (and occasionally membrane-bound) ligands of the EGF/neuregulin type and receptor tyrosine kinases of the EGFR family (Marchionni, 2014; Perrimon et al., 2012). Ligand binding induces receptor dimerization and cross-phosphorylation, activating downstream cascades such as the Erk-like mitogen-activated protein kinase (MAPK) and Akt-PI3K-mTOR pathways (Burgess, 2022). In the free-living flatworm *Schmidtea mediterranea*, EGF signalling is a key regulator of neoblast self-renewal and differentiation (Lei et al., 2016). This species expresses an EGF-like ligand, *nrg-7*, and a corresponding receptor, *egfr-3*, which together govern asymmetric neoblast division into both differentiating and self-renewing cells. Notably, in the acoel *Hofstenia miamia*, Stevens et al. (2025) recently identified a wound-induced upregulation of a neuregulin-like ligand, *nrg-1*, whose product likely interacts with a cognate EGF receptor, EGFR-1, to recruit neoblasts to the wound site. In *Echinococcus*, earlier work identified at least one EGFR-like receptor, termed EmER (Spiliotis et al., 2003), as well as downstream components of an Erk-like MAPK cascade (Spiliotis et al., 2006; Gelmedin et al., 2010). Cheng et al. (2017) further demonstrated that germinative cell proliferation is stimulated by exogenous human EGF and that metacestode growth is inhibited by candidate EGFR inhibitors such as afatinib, as well as by inhibitors of the Erk-like MAPK cascade. In a subsequent study, the same group showed that EGFR inhibitors induce apoptosis in germinative cells (Cheng et al., 2020), underscoring that, as in planarians, EGF signalling in cestodes is central to stem cell differentiation and self-renewal.

Although Cheng et al. (2017) found that host-derived EGF can activate the parasite EGFR EmER *in vitro*, clear effects were only observed at concentrations of 10 ng/ml or higher, which exceed physiological EGF levels in human tissues (1–2 ng/ml; Pinilla-Macua et al., 2017). Albeit hepatic EGF expression increases during liver regeneration (Mullhaupt et al., 1994), it remains unclear whether host-derived EGF reaches concentrations sufficient to drive parasite development *in vivo*. Moreover, free-living flatworms and acoels express several intrinsic EGF-like ligands (Barberan et al., 2016; Stevens et al., 2025), suggesting that *E. multilocularis* may also produce its own ligands to regulate stem cell proliferation. In this study, we aimed to obtain a comprehensive view of EGF signalling in *E. multilocularis*, encompassing the full repertoire of parasite EGF receptors, their expression patterns, functional relevance for parasite development, and the identification of parasite-derived ligands. To this end, we first characterize the three EGF receptors encoded in the parasite genome, then assess their roles using RNA interference and pharmacological inhibition, and finally identify and functionally analyze endogenous EGF-like ligands that interact with these receptors.

## Materials and methods

### Organisms and culture methods

All inhibitor, RNAi, and *in situ* hybridization experiments were performed with two isolates of *E. multilocularis*, H95 from a naturally infected fox (*Vulpes vulpes*) (Jura et al., 1996) and GH09 from a naturally infected cynomolgus monkey (*Macaca fascicularis*) (Tappe et al., 2007). At the time point of these experiments, GH09 was still capable of brood capsule and protoscolex formation, thus serving as the isolate used for WISH. H95 had lost the capacity to form protoscoleces, thus producing larger metacestode vesicles in culture that served for inhibitor experiments and yielding sufficient amounts of primary cells for RNA interference and primary cell culture. The isolates have been passaged in Mongolian jirds (*Meriones unguiculatus*) as described (Spiliotis et al., 2004; Spiliotis and Brehm, 2009). Preparation and use of conditioned media for axenic culture were carried out as previously described (Koike et al., 2022). Primary cell cultures had been set up essentially as previously described (Herz et al., 2024) and served as a cellular source for RNA interference experiments (Spiliotis et al., 2010) and for metacestode development from parasite stem cells (Herz et al., 2024).

Yeast Two-Hybrid (Y2H) Gold strain from Clontech (*MATa, trp1-901, leu2-3, 112, ura3-52, his3-200, gal4Δ, gal80Δ, LYS2:: GAL1_UAS_–Gal1_TATA_–His3, GAL2_UAS_–Gal2_TATA_–Ade2, URA3:: MEL1_UAS_–Mel1_TATA_, AUR1-C MEL1*) used in yeast two hybrid experiments was passaged once a month on conventional YPD plates and kept at 4°C. Dropout medium and plates were prepared with Difco™ Yeast Nitrogen Base without Amino Acid (Becton Dickinson) and DO supplements (Clontech) following the manufacturer’s instructions.

### Anti-parasitic inhibitor assays

Afatinib, osimertinib (both from Cayman chemicals), and dacomitinib (abcr GmbH) were prepared as 10 mM stock solutions in DMSO and utilized at the indicated concentrations (100 nM – 100 µM) in inhibitor studies with DMSO alone as control. Primary cells for cell viability assay and vesicle regeneration assay were isolated from *in vitro* cultivated metacestode vesicles using a previously established protocol (Spiliotis et al., 2010). Primary cell unit definition and experimental procedure of cell viability and regeneration assay were described in (Koike et al., 2022). The respective experiments were performed in 3 technical replicates and 3 biological replicates. The number of newly regenerated vesicles was analyzed by Kruskal-Wallis tests followed by Dunn’s tests in Graphpad Prism 10.1.2 (Graphpad software). The procedure of the mature vesicle assay is described by Koike et al. (2022). In this experiment, 3 biological replicates were used. The percentage of the intact vesicles in the mature vesicle assay and that of cell viability were analyzed with one-way-ANOVA followed by Dunnett’s tests in Graphpad Prism 10.1.2 (Graphpad software).

### Nucleic acid isolation, cloning, sequencing, and *in vitro* synthesis of cRNA

RNA isolation from HEK293T cells was performed using a Trizol-based method as described (Hemer et al., 2014). From total RNA, complementary DNA (cDNA) was synthesized with oligo(dT)20 primer and Superscript IV reverse transcriptase (Invitrogen) following the manufacturer’s instruction. For constructs of HsEGF, this cDNA was used as the PCR template. For the constructs of HER1, pMK-RQ-ErbB1 was used as a PCR template. For other constructs of *Echinococcus*, pJG4–5 based yeast-two-hybrid library from Hubert et al. (2004) were used as PCR templates. PCR primers were purchased from Merck (Germany) and are listed in **Supplementary Table S1**. Purified DNA fragments were cloned into linearized plasmids and sequenced by Sanger Sequencing (Microsynth seqlab). Sequences of newly analyzed genes of this work have been deposited in the GenBank database and accession numbers are listed in **Supplementary Table S1**. Prior to synthesis of capped mRNA (cRNA), pSecTag2Hygro-based plasmids (EmER1, EmER2, EmER3) and pcDNA3.1-SER (Vicogne et al., 2004) were linearized by *Pme I* and pOBER plasmid including HER1 (Opresko and Wiley, 1990) was linearized by *Not I*. cRNA was synthesized from these vectors as templates using the T7 mMessage mMachine Kit (Ambion). After purification and quantification, the size of the RNA fragment and the lack of abortive transcripts were confirmed through gel electrophoresis.

### Expression of EGFRs in *Xenopus* oocytes

Oocytes of *Xenopus laevis* were surgically removed from animals and prepared for the microinjection, as described (Vicogne et al., 2004). Oocyte viability was evaluated by counting GVBD+ (germinal vesicle breakdown) oocytes without microinjection, after progesterone treatment. 60ng of *emer1*, *emer2*, *emer3*, *SER*, and human *HER1* cRNA were injected into oocytes. 8 or 24 hours after microinjection of cRNA, the oocytes were stimulated by 50nM recombinant human EGF or progesterone. GVBD was evaluated 15 hours after progesterone/EGF stimulation. The lysates of oocytes were purified by myc-IP and tyrosine phosphorylation of the recombinant proteins was detected by Western Blot with anti PY100 antibody. To evaluate the effect of inhibitors, oocytes with or without cRNA microinjection were treated with various concentrations of inhibitors, dacomitinib, afatinib, and osimertinib for 30 minutes before EGF/progesterone stimulation. All experiments were performed using 2–3 technical replicates. Percentages of GVBD-positive oocytes were statistically analyzed with one-way ANOVA with Dunnett’s multiple comparison tests in Graphpad Prism 10.1.2 (Graphpad software).

### *In situ* hybridization and 5-ethynyl-2’-deoxyuridine labeling

Fragments of the *em-nrg* cDNA were amplified from a pJG4-5-based yeast-two-hybrid library (Hubert et al., 2004) and cloned into pJET1.2 with CloneJET PCR Cloning Kit (Thermo Fisher Scientific). Primers used for cloning are listed in **Supplementary Table S1**. Digoxygenin (DIG)-labeled probes were synthesized from this pJet 1.2-based plasmid, purified, and quantified as described (Koike et al., 2022). *In vitro* cultivated metacestode vesicles for whole-mount *in situ* hybridization (WISH) were treated with 40 mM hydroxyurea (HU) for 7 days as described (Koziol et al., 2014) and recovered for 0, 3, 6, or 10 days. All vesicles were labelled with 50 μM 5-ethynyl-2’-deoxyuridine (EdU) *in vitro* at 37°C for 5 h prior to fixation by 4% paraformaldehyde. WISH followed by fluorescent detection of EdU with Alexa Flour 555 was carried out as previously described (Koziol et al., 2014; Herz et al., 2024). Fluorescent specimens were imaged with a Nikon Eclipse Ti2E confocal microscope and processed with Fiji/ImageJ as described (Koike et al., 2022). One way ANOVA followed by Dunnett’s multiple comparison test was used in Graphpad Prism 10.1.2 (Graphpad software) to compare the number of cells with each signal. Pulse-chase experiments were carried out essentially as recently described (Herz et al., 2024) with a 5 h EdU pulse followed by 72 h recovery prior to *emer1*-specific WISH and double staining.

### Membrane-anchored ligand and receptor yeast two-hybrid (MALAR Y2H) analyses

The previously established MALAR-Y2H system (Li et al., 2016) was used for assessing ligand-receptor interactions, with plasmids pGAD_SP-WBP1_cloning_ linker_TMP_Cub_GAL4 (for ligands), and pGBKT7_SP_OST1_cloning_NubG (for receptors). cDNA fragments encoding sequences downstream of the signal peptide and upstream of the transmembrane domain of the *Echinococcus* ligand cDNA were amplified from pJC4–5 based plasmid library (Hubert et al., 2004), and integrated into *SmaI*-digested pGAD_SP-WBP1_cloning_linker_TMP_Cub_GAL4. For the human EGF construct, cDNA synthesized from HEK293T cells was used for the PCR template. Similarly, fragments between signal peptide and juxtamembrane region of the receptor cDNA were amplified and integrated into *SmaI*-digested pGBKT7_SP_OST1_cloning_NubG. Primers used for cloning are listed in **Supplementary Table S1**. The *Saccharomyces cerevisiae* Y2HGold strain (Clontech) was transformed by these plasmids using the protocol of Tripp et al. (2013). Transformants were initially inoculated onto Leu-/Trp- double dropout plates, and later transferred onto Leu-/Trp-/His- triple dropout plate and Leu-/Trp-/Ade-/His- quadruple dropout plates with three densities, as described (Koike et al., 2022). Growth level was quantified by Fiji/ImageJ using the protocol of Petropavlovskiy et al. (2020). The quantified level of growth on quadruple dropout plates was statistically analyzed using one-way-ANOVA followed by Tukey’s multiple comparison tests in Graphpad Prism 10.1.2 (Graphpad software).

### RNA interference

RNA interference (RNAi) was performed using a slightly modified protocol of Spiliotis et al. (2010). siPOOLs (mixture of 30 siRNA fragments) were designed and synthesized by siTOOLs Biotech (Planegg, Germany). Other siRNAs were designed with siDirect version 2.0 (Naito et al., 2009) and purchased from Sigma-Aldrich. As negative and positive controls for siPOOLs, randomized siRNA fragments and fragments against the *bcat-1* gene (Herrmann et al., 2025) were used, respectively. As negative and positive controls for siRNA (Sigma), siRNA against eGFP and *bcat-1* were used, respectively. The sequences of the siRNAs are listed in **Supplementary Table S1**. *E. multilocularis* primary cells for RNAi experiments were isolated with the same procedure as those for cell viability assay and vesicle regeneration assay. 150 U of primary cells isolated from mature metacestode vesicles were dispensed into an electroporation cuvette (1 mm Gap) from BTX with 90 µl of electroporation buffer (siPort) including 200 pmol siRNA fragments and electroporated with time constant protocol at 200 V for 0.5 ms with the electroporator GenePulser Xcell (BioRad). The electroporated primary cells were incubated for 12 min at 37°C and centrifuged at room temperature. After removal of the supernatant, cells were resuspended by the A6/B4 condition medium and dispensed into a 96-well plate (150U/well). Primary cells were then cultured for 21 days, and half of the conditioned medium was exchanged twice a week. Images were taken under an optical microscope (Nikon eclipse Ts2-FL) and the number of regenerated vesicles was counted. Three technical replicates and three biological replicates were prepared for counting regenerated vesicles. The numbers of regenerated vesicles were analyzed by Kruscal-Wallis tests followed by Dunn’s tests in Graphpad Prism 10.1.2 (Graphpad software). With only three technical replicates, cell viability was measured with Cell Titer Glo (Promega) after 21 days of incubation.

### Bioinformatic analysis

Bioinformatic analysis was essentially performed as described (Koike et al., 2022). Briefly, BLASTP searches against protein databases of *E. multilocularis*, *Schistosoma mansoni*, and *Schmidtea mediterranea* were performed on Wormbase ParaSite (Howe et al., 2017) using the amino acid sequences of human EGF receptors and EGF ligands as queries. The KEGG database at Genomenet (https://www.genome.jp/) was used for the reciprocal BLASTP analysis. Gene predictions based on the *E. multilocularis* genome sequence (Tsai et al., 2013), as available in Wormbase ParaSite, were verified or corrected using the Integrated Genome Viewer (Thorvaldsdóttir et al., 2012) and previously generated transcriptome data (Tsai et al., 2013; Herz et al., 2024). For domain detection, SMART (Letunic et al., 2021) was used. Multiple sequence alignments were performed using CLUSTALW 2.1 (Thompson et al., 1994). Statistical analyses were performed using Graphpad Prism 10.1.2 throughout. Statistical comparisons were performed only where biologically meaningful reference groups were available (e.g., treated versus control conditions). For datasets describing gene expression levels across different developmental stages without a defined control condition, no statistical testing was applied, as such comparisons would not be biologically informative.

## Results

### Cloning and characterization of *Echinococcus* EGF receptors

To systematically dissect EGF signalling in *E. multilocularis*, we first identified and molecularly characterized the parasite’s EGF receptor repertoire before assessing receptor function, pharmacological inhibition, and ligand–receptor interactions. One receptor of this family, EmER, had previously been described and shown to exhibit the typical EGFR architecture: an intracellular tyrosine kinase domain and two extracellular receptor L domains accompanied by several furin-like repeats (Spiliotis et al., 2003). This structural combination is a hallmark of the EGF and insulin receptor families (Ward et al., 2007).

Inspection of the annotated *E. multilocularis* genome sequence (Tsai et al., 2013) identified only five gene models encoding proteins with this composition. Two (EmuJ_000962900, EmuJ_000981300) belonged to the insulin receptor family, which we had characterized previously (Konrad et al., 2003; Hemer et al., 2014); one corresponded to EmER (EmuJ_000075800); and two others (EmuJ_000617300, EmuJ_000969600) were annotated as receptor tyrosine kinases. BLASTP analyses revealed that the latter proteins showed the highest similarity to EGF receptors from other metazoans. We therefore concluded that EmuJ_000617300 and EmuJ_000969600 represent two additional EGF receptor genes, consistent with previous analyses of EGFR evolution in metazoans (Barberan et al., 2016).

To ensure that no further genes had been overlooked, we carried out TBLASTN searches against the genome using the tyrosine kinase and receptor L domains of EmuJ_000075800, EmuJ_000617300, and EmuJ_000969600 as queries. No additional candidates were identified. Thus, in contrast to planarians, which encode six EGF receptor genes (Barberan et al., 2016), the *E. multilocularis* genome contains only three. Guided by the genome sequence and recent transcriptome data (Herz et al., 2024), we designed primers to clone the remaining EGF receptors in full length and deposited the cDNA sequences in GenBank (**Supplementary Table S1**).

Nomenclature has been inconsistent in the literature. The first cloned receptor, corresponding to EmuJ_000075800, was originally named *emer* (Spiliotis et al., 2003), but Barberan et al. (2016) referred to it as EGFR-2, designating EGFR-1 for EmuJ_000617300 and EGFR-3 for EmuJ_000969600. In earlier genome analyses, we named these genes *emerb* and *emerc* (Brehm and Koziol, 2017). To harmonize with established conventions for naming insulin (Hemer et al., 2014), fibroblast growth factor (Förster et al., 2019), and transforming growth factor (Kaethner et al., 2023) receptors, we now designate them *emer1* (EmuJ_000075800), *emer2* (EmuJ_000617300), and *emer3* (EmuJ_000969600), encoding EmER1, EmER2, and EmER3, respectively. Notably, while the cDNAs for EmER1 and EmER3 were correctly predicted in genome sequencing (Tsai et al., 2013), the annotation of EmER2 was incomplete: the true cDNA encodes an additional 31 N-terminal amino acids, including a signal peptide.

**Figure 1:**
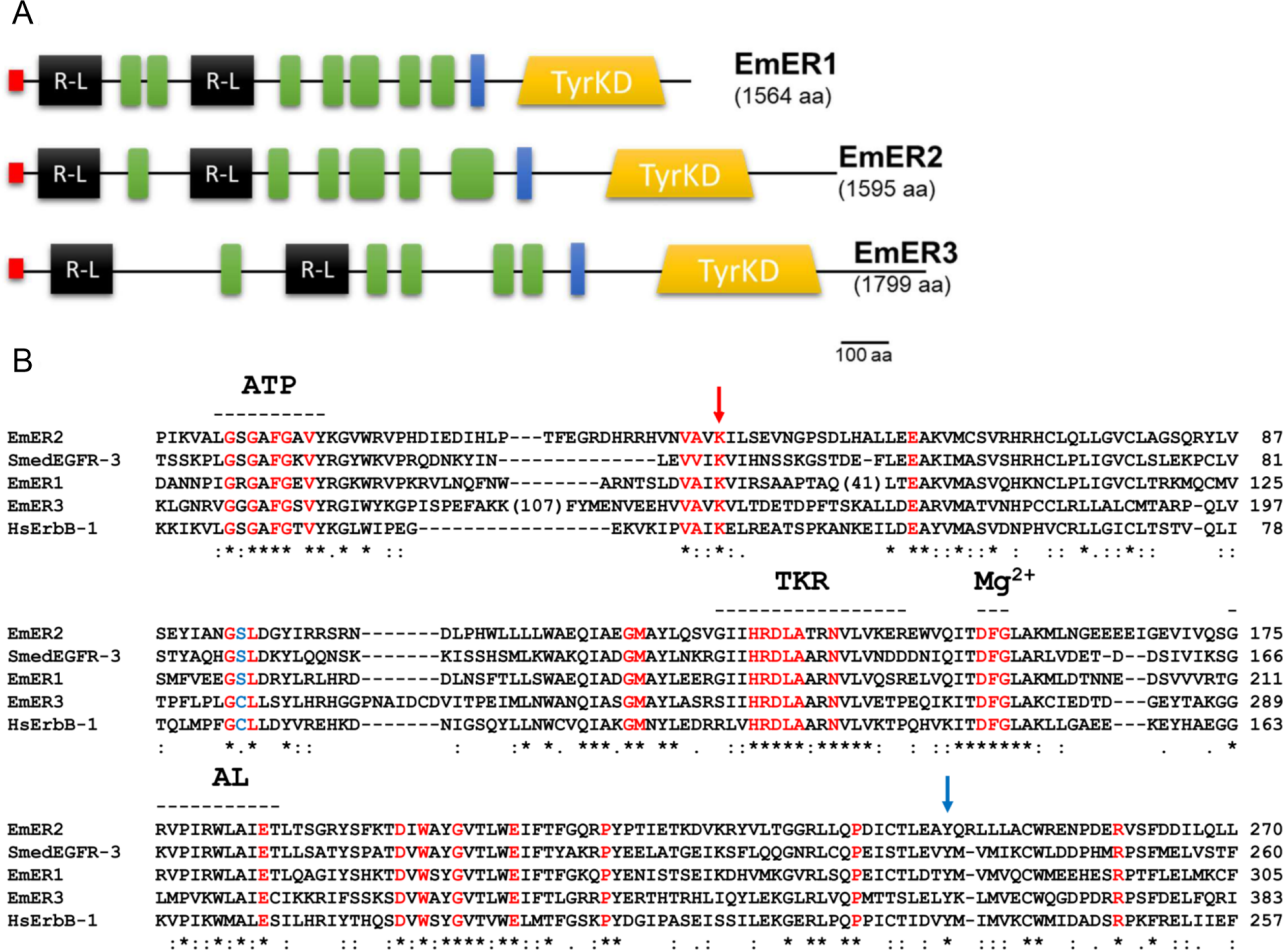
Domain structure and homologies of *E multilocularis* EGF receptors. **(A)** Domain structure of *E multilocularis* receptors EmER1, EmER2, and EmER3. Shown are the positions of the following domains: tyrosine kinase (TyrKD, yellow), transmembrane domain (blue), receptor-L-domains (R-L, black), furin-like repeats (green), and N-terminal signal peptides (red). Numbers in brackets indicate full-length amino acids. Size bar indicates 100 amino acids. **(B)** Amino acid sequence comparison of the tyrosine kinase domains of *E multilocularis* EGF receptors, human ErbB-1, and *S. mediterranea* EGFR-3 (as indicated). Amino acid residues that are highly conserved among receptor tyrosine kinases (Hanks et al., 1988) are marked in red. The position of a Cys-residue of ErbB1 that covalently binds afatinib is marked in blue. The locations of the ATP-binding motif (ATP), the catalytic loop (TKR), the Mg^2+^ binding site (Mg2^+^), and the activation loop (AL) are indicated above the alignment. A red arrow marks the catalytic lysin, a blue arrow a tyrosine that is phosphorylated for autoactivation. Code below the alignment indicates invariant residues (*) as well as strongly (): and weakly (.) conserved residues. Numbers in brackets indicate the length of insert sequences that do not align with other receptors.

As shown in **Figure 1A**, all three *Echinococcus* EGF receptors possess predicted tyrosine kinase domains in their cytoplasmic regions, as well as signal peptides, two receptor L domains, and several furin-like domains extracellularly. In EmER2, the SMART algorithm (Letunic et al., 2020) did not detect a transmembrane helix; however, Kyte–Doolittle hydropathy analysis (Kyte and Doolittle, 1982) identified a strongly hydrophobic stretch between residues 970 and 1000, which we propose constitutes the transmembrane region. Sequence alignments of the tyrosine kinase domains further confirmed that all three receptors retain the canonical residues required for enzymatic activity (**Figure 1B**), indicating that they function as active tyrosine kinases.

### Expression patterns of *Echinococcus* EGF receptor genes

**Figure 2:**
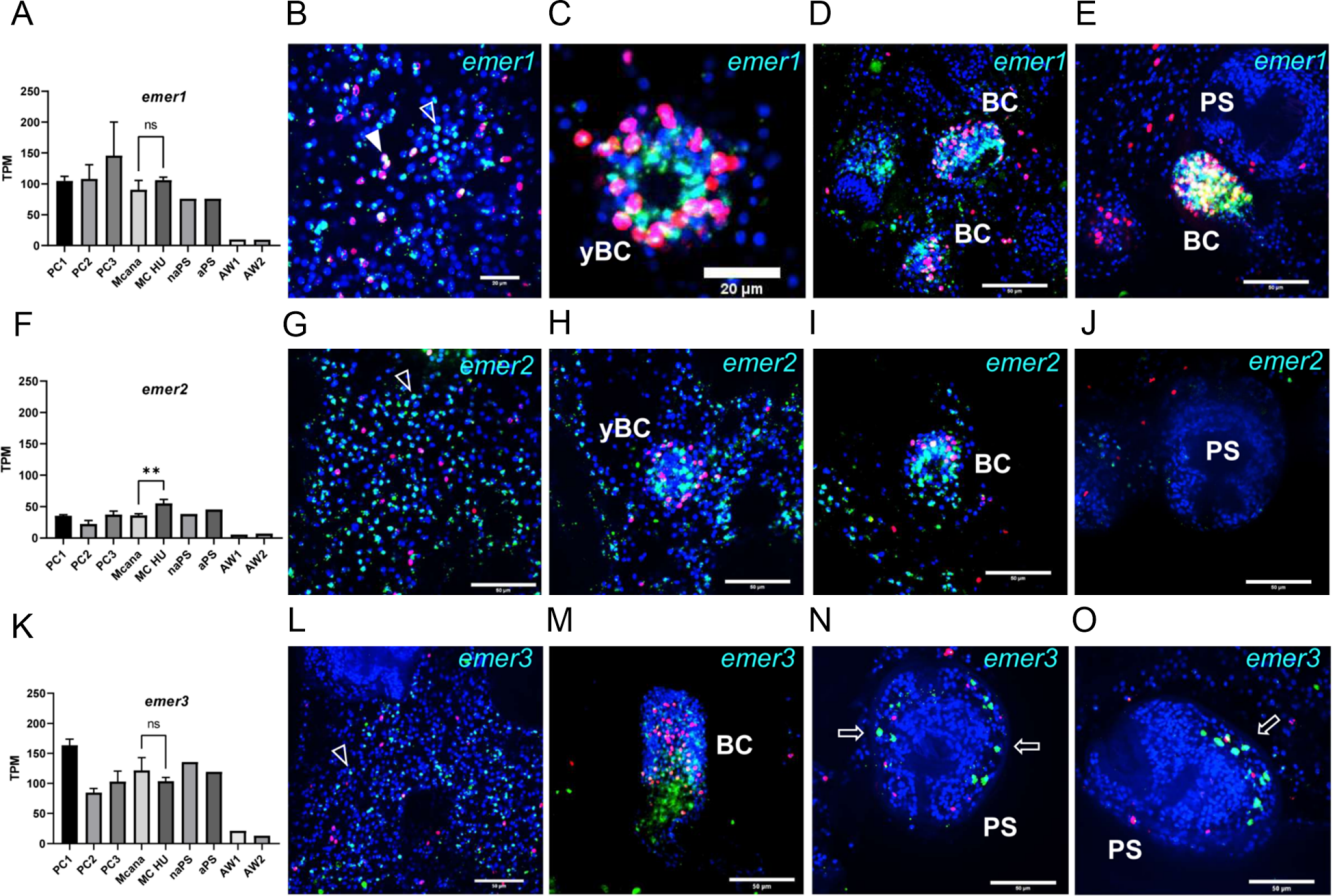
Expression of EGF receptor genes in *Echinococcus* larvae. **(A, F, K)** Expression values (in TPM) of *Echinococcus* EGF receptor encoding genes *emer1***(A)**, *emer2***(F)**, and *emer3***(K)** in primary cell cultures after 2 (PC1), 7-11 (PC2), and 16–22 days (PC3), *in vitro* cultivated metacestode vesicles before (MCana) or after (MC HU) treatment with hydroxyurea, non-activated (naPS) and activated (aPS) protoscoleces as well as pre-gravid (AW1) and gravid (AW2) adult worms (all according to Herz et al., 2024 and Tsai et al., 2013). Expression values represent transcript abundance across developmental stages. No statistical comparisons were performed, as no defined control or treatment groups were present, except for MCana versus MC-HU [unpaired t-test, p = 0.0076 (**)]. **(B-E)** WISH for *emer1* in germinal layer **(B)**, young **(C)**, advanced **(D)**, and older **(E)** brood capsules. **(G-J)** WISH for *emer2* in germinal layer **(G)**, young **(H)** and advanced **(I)** brood capsules as well as dormant protoscolex **(J, L-O)** WISH for *emer3* in germinal layer **(L)**, advanced brood capsule **(M)**, and protoscolex **(N,O)**. WISH+ cells are indicated by open triangle, WISH+/EdU+ cells by closed triangle. Open arrows indicate lateral *emer3* expression in protocolex. Shown are merge images (single confocal slices) of all three channels (blue, DAPI, nuclei; red, EdU, S-phase stem cells; green, WISH). BC, brood capsule; yBC, young brood capsule; PS, protoscolex. Scale bar represents 50 µm in all slides except C (20 µm).

We investigated the expression profiles of *emer1*, *emer2*, and *emer3* across different developmental stages of *Echinococcus*. To this end, we analyzed previously generated transcriptome datasets covering protoscoleces (before and after activation by low pH/pepsin treatment) and adult worms (Tsai et al., 2013), as well as metacestode vesicles (before and after hydroxyurea treatment to deplete stem cells) and parasite primary cell cultures at various developmental stages (Herz et al., 2024). As shown in **Figure 2**, all three genes were expressed in metacestode vesicles, with *emer2* consistently exhibiting the lowest overall expression levels. A similar pattern was observed in protoscoleces, where *emer2* again displayed the weakest expression. Importantly, none of the EGFR-encoding genes showed significant reductions in transcript abundance after hydroxyurea treatment of metacestode vesicles, a feature we previously identified as characteristic of stem cell-associated genes (Herz et al., 2024). This suggests that *emer1*, *emer2*, and *emer3* are expressed predominantly in differentiated or differentiating cells. By contrast, expression was comparatively low in adult worms. Examination of a transcriptome dataset for oncospheres (Huang et al., 2016) revealed substantial expression only for *emer1* and *emer3*. Overall, *emer1* and *emer3* are prominently expressed throughout the parasite life cycle, whereas *emer2* appears to be more weakly transcribed.

To further define the expression patterns of *Echinococcus* EGFR genes, we performed WISH on *in vitro* cultivated metacestode vesicles, in combination with EdU staining (5 h pulse) to label S-phase stem cells in metacestode vesicles without brood capsules. As shown in **Figure 2**, all three genes produced numerous signals distributed across the germinal layer. In the case of *emer1*, we counted 18.1% (± 0.8%) of all cells positive in WISH with 7.1% (± 0,4) EdU+ cells and 1.7% (± 0.1) cells that stained both positive for EdU and for WISH (n = 8405). For *emer2*, we counted 27.5% (± 4,0) as WISH+, 7.4% (± 0.5) as EdU+ and less than 0.5% positive for both (n = 8374). For *emer3* we counted 22.0% (± 3.5) WISH+, 7.8% (± 0.7) EdU+ and, again, less than 0.5% positive for both signals (n = 8395). Together, these data indicate that all three genes are predominantly expressed in post-mitotic cells that are either differentiated or differentiating and that *emer1* is the only gene that, to a certain extent, is also expressed in S-phase stem cells. It is interesting to note that *emer2*, albeit showing the lowest expression level of all three genes in transcriptomic analyses (Herz et al., 2024), stains positive for overall more cells within the germinal layer. This indicates that *emer1* and *emer3* are expressed to a relatively high level within the WISH+ cells.

We then also carried out WISH in vesicles that produced brood capsules and protoscoleces. As depicted in **Figure 2**, we obtained strong signals for *emer1* in young and developing brood capsules (**Figures 2C–E**), whereas no signal was detected in the non-activated protoscolex within vesicles (**Figure 2E**). Likewise, we found *emer2* expressed in young and developing brood capsules (**Figures 2H, I**) but no signal in the fully developed, dormant protoscolex (**Figure 2J**). For *emer3*, we observed a strong signal at the base of developing brood capsules (**Figure 2M**) and, later, a striking expression pattern in lateral cells of the protoscolex, (**Figures 2N,O**) suggesting a role in protoscolex pattern formation.

In summary, our analyses demonstrate that all three *Echinococcus* EGFR genes are highly expressed in the metacestode stage, predominantly in post-mitotic cells, and are likely to play roles in protoscolex development.

### *emer1* is essential for metacestode formation

**Figure 3:**
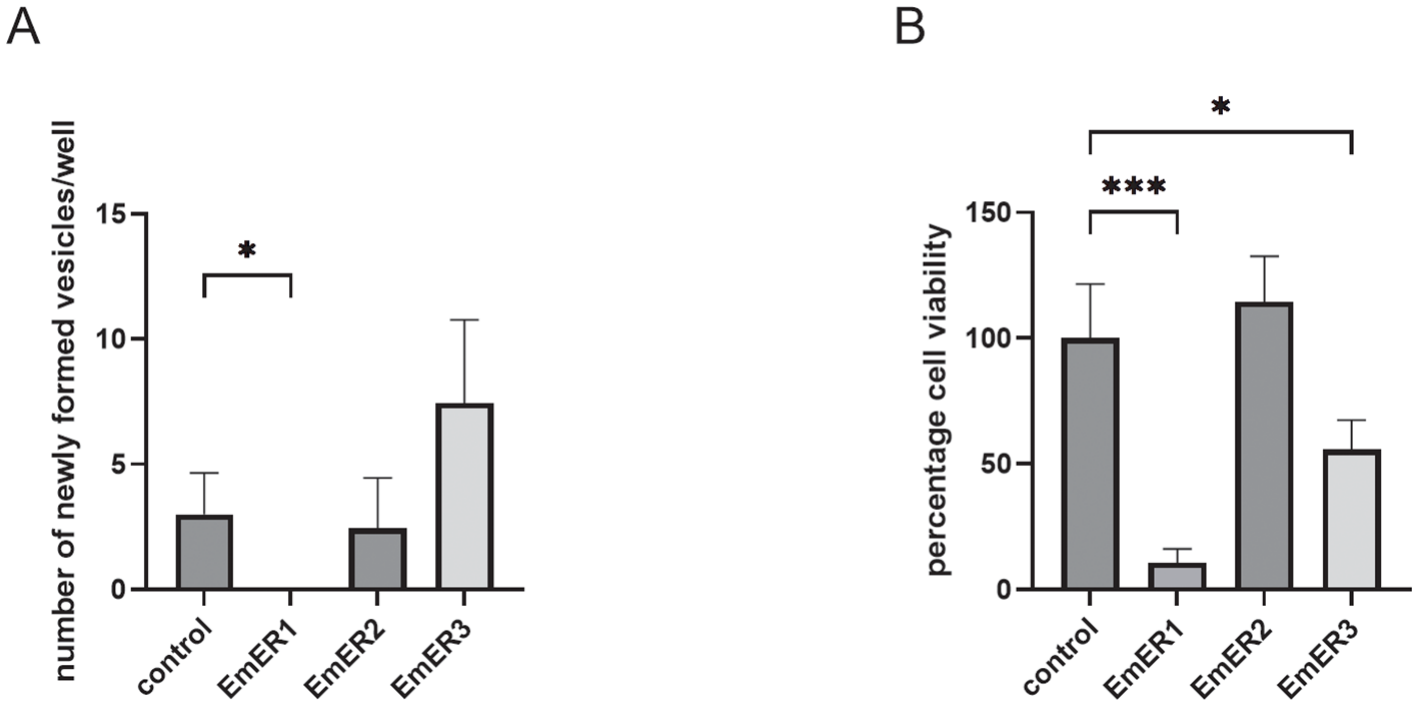
*emer1* is necessary for metacestode development. Displayed are the results of RNAi knockdown of genes encoding EmER1, EmER2, and EmER3 (as indicated). **(A)** Formation of mature metacestode vesicles from primary cell cultures after 21 days. The numbers of regenerated vesicles were analyzed by Kruscal-Wallis tests followed by Dunn’s tests. P values less than 0.0332 were summarized with *. **(B)** Cell viability of primary cell cultures after 3 days. Experiments have been performed as three biological triplicates. Normalized cell viability was analyzed by one-way ANOVA followed by Dunnet’s test. Error bar represents standard deviation. P values less than 0.0332, and 0.0002 are summarized with * and ***, respectively.

We previously established a method for silencing *Echinococcus* genes in primary cell cultures using RNA interference (RNAi) (Spiliotis et al., 2010). In this system, primary cell cultures that are enriched in parasite stem cells (Koziol et al., 2014) are set up from *in vitro* cultivated metacestode vesicles and typically yield mature metacestode vesicles due to stem cell proliferation and differentiation after about 2–3 weeks (Koziol et al., 2014; Brehm and Koziol, 2017; Herz et al., 2024). The primary cell culture system is currently also the only system for functional, RNAi-based studies on metacestode-expressed genes in Echinococcus (Spiliotis et al., 2010; Herrmann et al., 2025). Since all three EGFR encoding genes are well expressed in parasite primary cells (**Figure 2**), we examined their roles in metacestode development by combining our RNAi protocol with siPOOL technology (Hannus et al., 2014) to minimize off-target effects. Using this strategy, transcript levels were reduced by approximately 60% for each gene three days after RNAi application (**Supplementary Figure S1**). We then monitored vesicle formation over 21 days. Notably, *emer1*(RNAi) cultures failed to generate mature metacestode vesicles, whereas *emer2*(RNAi) had no detectable effect and *emer3*(RNAi) even enhanced vesicle formation (**Figure 3A**). To investigate the cellular basis of the *emer1*(RNAi) phenotype, we assessed primary cell viability (Koike et al., 2022) following knockdown. These experiments revealed significantly reduced viability in *emer1*(RNAi) cultures, while *emer2*(RNAi) and *emer3*(RNAi) cultures remained unaffected (**Figure 3B**). Collectively, these findings demonstrate that *emer1* is indispensable for metacestode vesicle formation and that its loss compromises parasite cell viability.

### Afatinib targets EmER1

Cheng et al. (2020) reported that treatment with afatinib (BIBW2992) at concentrations around 5 µM disrupts the structural integrity of metacestode vesicles *in vitro*, induces apoptosis in germinative cells, and reduces parasite burden in infected mice. Thus, afatinib is currently the most promising EGFR inhibitor with potential for repurposing against AE. However, the precise *Echinococcus* EGFR targeted by afatinib has remained unknown. To address this, and to further evaluate the effects of afatinib, we conducted comparative analyses including several related EGFR inhibitors.

We first tested the effects of inhibitors on isolated parasite primary cells for cell viability and metacestode vesicle regeneration capacity as well as on mature metacestode vesicles, according to a recently conducted study on anti-parasitic activities of PIM kinase inhibitors (Koike et al., 2022). As shown in **Supplementary Figure S2**, multiple EGFR inhibitors showed strong effects against primary cells in the screening. For further analysis, we therefore chose afatinib and dacomitinib (second-generation EGFR receptors) together with osimertinib as a representative of the third generation.

**Figure 4:**
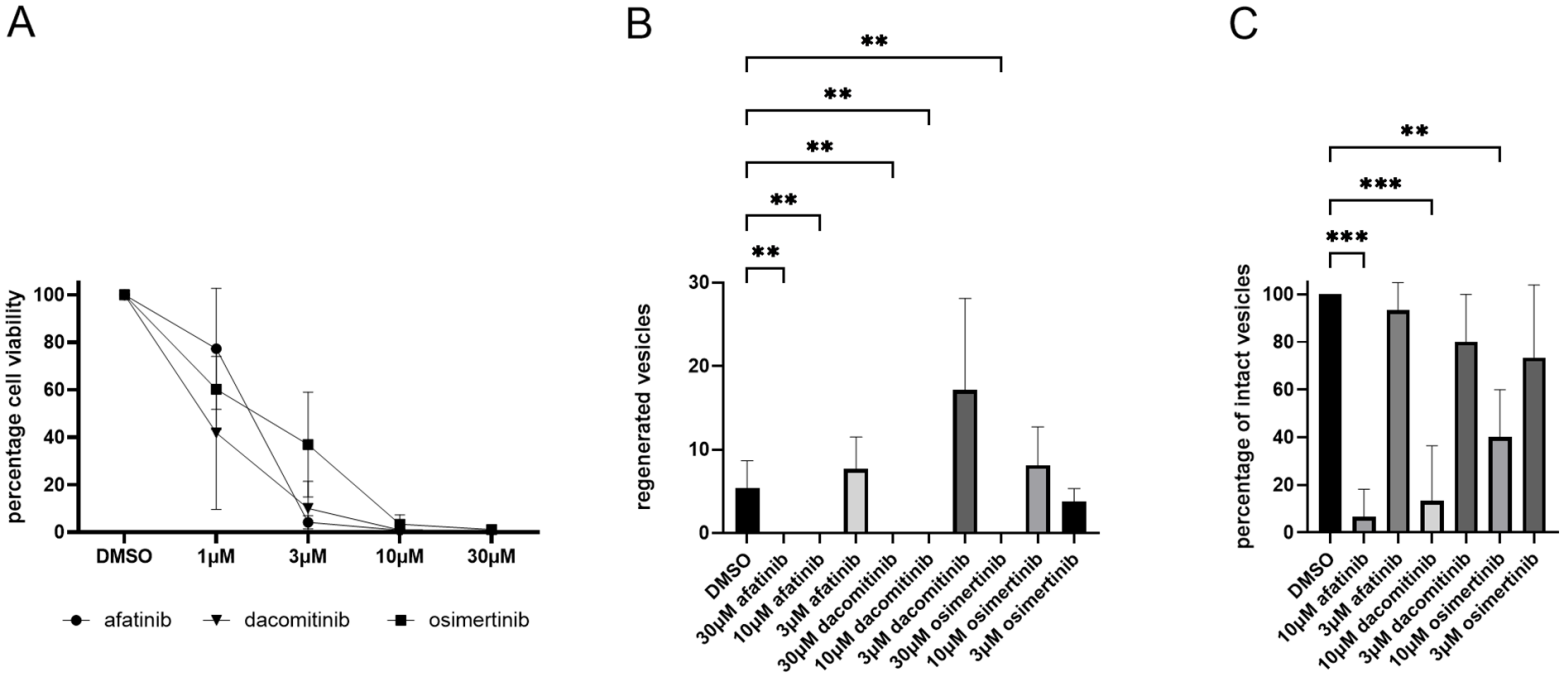
Effects of EGF receptor inhibitors on *Echinococcus* primary cells and metacestode vesicles. **(A)** Effects of afatinib, dacomitinib, and osimertinib on primary cell culture viability after 3 days of cultivation. Inhibitor concentrations are indicated. **(B)** Effects of EGF receptor inhibitors on the regeneration of metacestode vesicles from primary cell cultures. Cell cultures were incubated in the presence of inhibitors (concentration as indicated below) for 21 days and regenerated vesicles were counted. **(C)** Effects of inhibitors on metacestode vesicle structural integrity after 28 days. Inhibitor concentrations are indicated below. DMSO was used as negative control. Error bar indicates standard deviation. p values less than 0.0021 and 0.0002 are indicated by ** and ***, respectively.

We then tested the compounds on isolated parasite primary cells, assessing both cell viability and vesicle regeneration capacity, as well as on mature vesicles, following protocols previously applied to PIM kinase inhibitors (Koike et al., 2022). As shown in **Figure 4**, afatinib, dacomitinib, and osimertinib (1–10 µM) all reduced the viability of primary cells, with afatinib exerting the strongest effects. At 10–30 µM, afatinib and dacomitinib completely blocked vesicle regeneration, while osimertinib achieved this only at 30 µM. Interestingly, dacomitinib at 3 µM even stimulated vesicle regeneration (**Figure 4B**). All three inhibitors also compromised vesicle integrity *in vitro*, again with afatinib being the most potent (**Figure 4C**). These results corroborate the observations of Cheng et al. (2020) and additionally identify dacomitinib and osimertinib as EGFR inhibitors with anti-*Echinococcus* activity, although less effective than afatinib.

**Figure 5:**
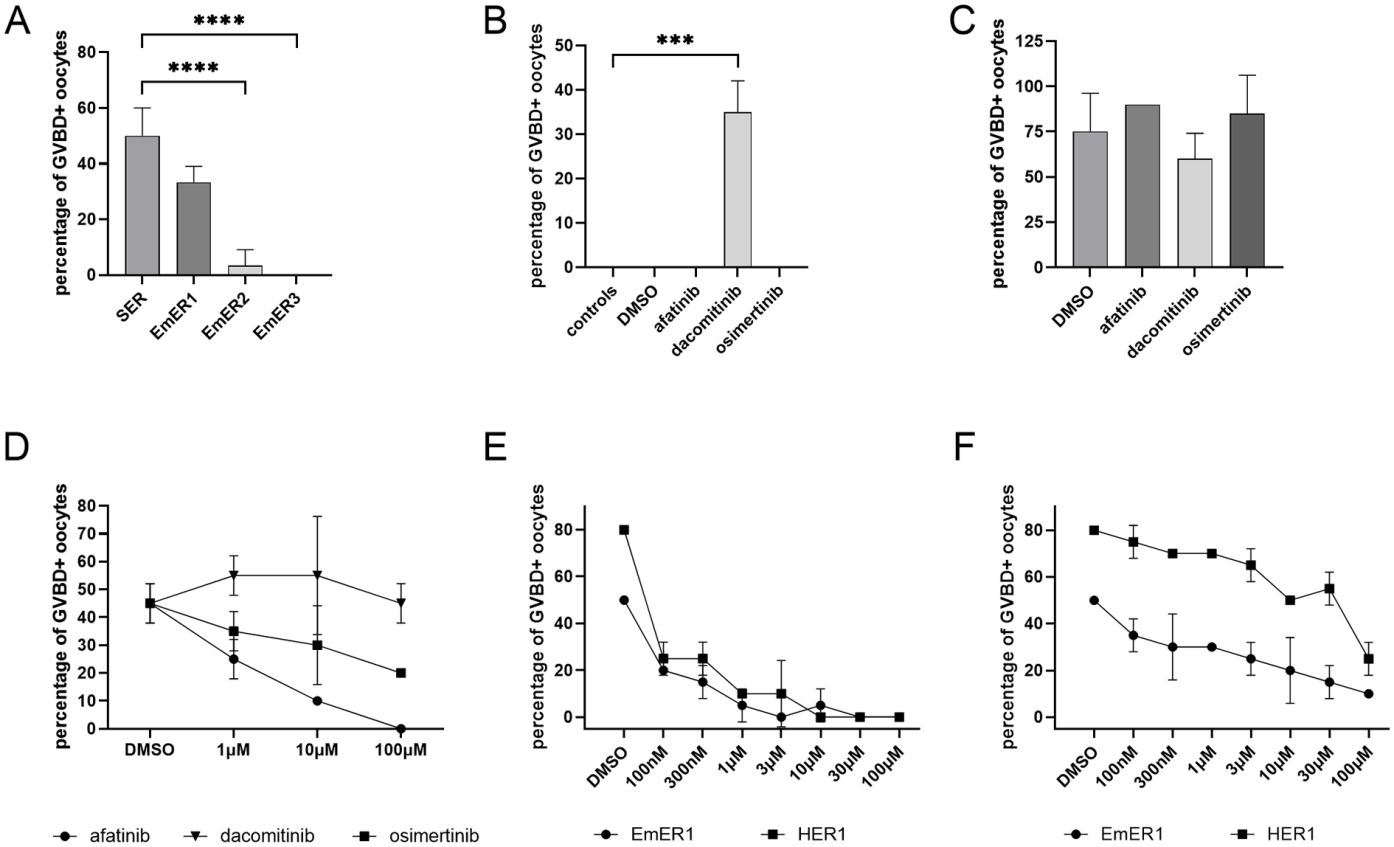
Effects of inhibitors on recombinantly expressed *Echinococcus* EGF receptors. cRNAs encoding EmER1, EmER2, EmER3, human HER1, and the *S. mansoni* receptor SER were expressed in *Xenopus* oocytes and incubated in the presence of EGF inhibitors as indicated. Subsequently, germinal vesicle breakdown (GVBD) was measured. **(A)** Expression of parasite receptors (as indicated below) in *Xenopus* oocytes and stimulation by 50 nM human EGF. GVBD was measured after 15 h incubation. 3 technical replicates were used. Error bar represents standard deviation. p values less than 0.0001 are indicated by ****. **(B)***Xenopus* oocytes were incubated in the presence of 10 μM inhibitors for 30 min and GVBD was measured after 15 h incubation. 2 technical replicates were used. Error bar represents standard deviation. p values less than 0.0002 are indicated by ***. **(C)** Percentages of GVBD+ oocytes are shown. Oocytes were treated with 10μM of inhibitors for 30 minutes and subsequently stimulated by progesterone. GVBD+ oocytes were counted 15 hours after progesterone stimulation. 10 oocytes were used for each condition with 2 technical replicates. Error bars represent standard deviation. **(D)** Percentages of GVBD+ oocytes are shown. 8 hours after microinjection of EmER1 encoding cRNA, oocytes were treated with 100 nM, 1 μM and 10 μM of inhibitors for 30 minutes and stimulated by 50 nM human EGF. GVBD+ oocytes were counted 15 h after stimulation by human EGF. 10 oocytes were used for each condition with 2 technical replicates. Error bars represent standard deviation. **(E, F)** Percentages of GVBD+ oocytes are shown. 8 hours after cRNA microinjection encoding EmER1 and HER1, oocytes were treated with 100 nM, 300 nM, 1 μM, 3 μM, 10 μM and 100 μM of afatinib **(E)** or osimertinib **(F)** for 30 min and stimulated by human EGF. GVBD+ oocytes were counted 15 h after EGF stimulation. 10 oocytes were used for each condition with 2 technical replicates. Error bars represent standard deviation.

To identify the molecular target of afatinib, we employed the *Xenopus* oocyte system, previously used to assess flatworm EGFR activity (Vicogne et al., 2004). As ligand stimulation is required in this assay, we used human EGF (HsEGF) based on prior work (Cheng et al., 2017) and included the *S. mansoni* receptor SER as a positive control. As shown in **Figure 5A**, EmER1 expression combined with HsEGF stimulation induced 30% GVBD, comparable to SER (50%), confirming that EmER1 is an active kinase responsive to HsEGF. By contrast, EmER2 and EmER3 elicited little to no GVBD, suggesting they are either inactive kinases or do not interact with HsEGF. Expression and phosphorylation assays in oocytes further supported this conclusion: all receptors were expressed, but phosphorylation was only observed for EmER1 upon HsEGF stimulation (**Supplementary Figure S3**).

We next tested afatinib and osimertinib on EmER1 activity in *Xenopus* oocytes. Importantly, progesterone-induced GVBD remained intact in the presence of both inhibitors, excluding general toxicity (**Figure 5C**). Dacomitinib was not pursued due to non-specific GVBD induction (**Figure 5B**). Both afatinib and osimertinib inhibited EmER1 in a dose-dependent manner, with ~50% inhibition already at 1 µM (**Figure 5D**). Further titration from 100nM to 100 μM revealed that afatinib was highly effective even at 100nM against both HER1 and EmER1, whereas osimertinib consistently showed weaker effects against HER1 than against EmER1 (**Figures 5E, F**).

Taken together, our data confirm afatinib’s profound activity against *Echinococcus* cells and vesicles, extend its efficacy to germinative cell-enriched cultures (~80%; Koziol et al., 2014), and identify EmER1 as its principal molecular target. These findings strongly suggest that the anti-parasitic effects of afatinib observed *in vitro* and *in vivo* (Cheng et al., 2020) are primarily mediated through inhibition of EmER1.

### Cloning and characterization of *Echinococcus* EGF-like ligands

Having identified EmER1 as a key regulator of metacestode development and the principal target of EGFR inhibitors, we next asked whether *E. multilocularis* encodes endogenous EGF-like ligands capable of activating this receptor. The defining feature of EGF-like ligands is the presence of an EGF domain with six characteristic cysteine residues forming disulfide bridges. However, such domains also occur in the extracellular regions of many unrelated transmembrane proteins (Wouters et al., 2005). In a previous bioinformatic survey, Barberan et al. (2016) applied stringent criteria for identifying bona fide EGF-like ligands in metazoans and specifically, the presence of a single EGF domain in combination with a transmembrane domain and, in the case of neuregulin subtypes, an immunoglobulin (IG) domain. Based on these criteria, they identified two potential *Echinococcus* EGF-like ligands. One, represented by gene model EmuJ_000753300 (EmEGF), was categorized as an EGF-type ligand, while the second (EmuJ_000090400; EmNRG) showed structural characteristics of the neuregulin family, which are recognized as cognate ligands of EGFRs (Marchionni, 2014). Guided by the *E. multilocularis* genome sequence (Tsai et al., 2013) and recently generated transcriptome data (Herz et al., 2024), we designed primers to clone the full-length cDNAs of both genes, which we designated *em-egf* (EmuJ_000753300) and *em-nrg* (EmuJ_000090400), encoding the proteins EmEGF and EmNRG, respectively.

**Figure 6:**
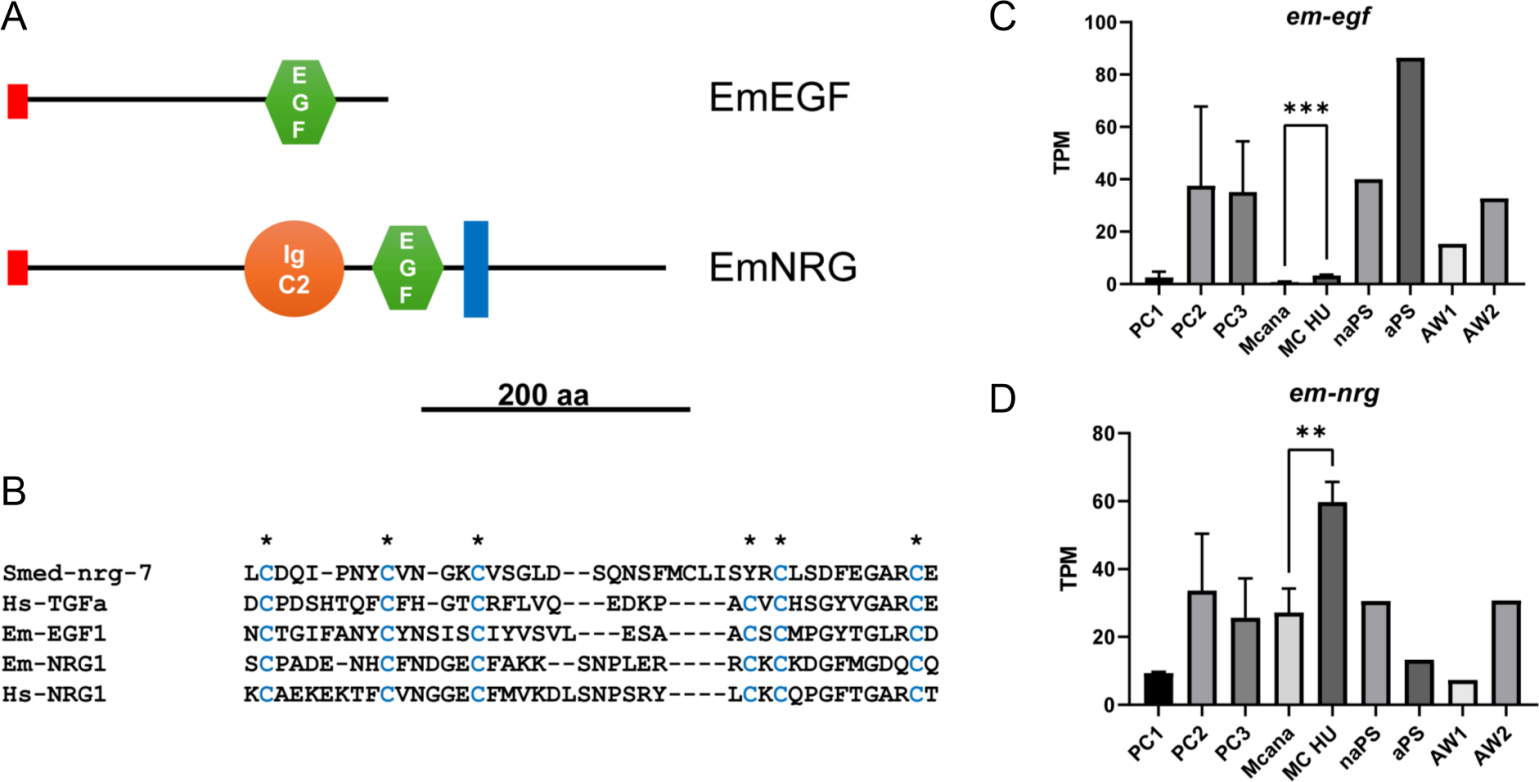
Structure and expression of *Echinococcus* EGF ligands. **(A)** Domain structure of EmNRG and EmEGF. Displayed are N-terminal signal peptide (red), immunoglobulin type C-2 domain (orange), EGF domain (green), and transmembrane region (blue). Scale bar represents 200 amino acids. **(B)** Amino acid sequence comparison between the EGF domains of EmNRG, EmEGF, human EGF ligands NRG1 and TGFα, as well as neuregulin-7 of *S. mediterranea*. Asterisks indicate 6 characteristic Cys residues (in blue) that form disulfide bridges. **(C, D)** Expression of *em-egf***(C)** and *em-nrg***(D)** in primary cell cultures at different time points of development (PC1 – PC3; see **Figure 2**), metacestode vesicles before (MC ana) or after (MC HU) treatment with hydroxyurea, non-activated (naPS) and activated (aPS) protoscoleces as well as pre-gravid (AW1) and gravid (AW2) adult worms. Expression values are in TPM and taken from Herz et al. (2024) and Tsai et al. (2013). Expression values represent transcript abundance across developmental stages. No statistical comparisons were performed, as no defined control or treatment groups were present, except for MCana versus MC-HU (unpaired t-test, p = 0.0037 (**), p = 0.0005 (***).

As illustrated in **Figure 6A**, both proteins possess N-terminal signal peptides and a single EGF domain. EmNRG also contains an immunoglobulin C-2 domain, a hallmark of the neuregulin subfamily of EGF-like ligands (Marchionni, 2014). Moreover, EmNRG harbors a transmembrane domain, suggesting either direct involvement in cell-cell communication or the requirement for proteolytic cleavage of its extracellular portion to generate a soluble ligand. In both proteins, the EGF domains feature six conserved cysteine residues responsible for disulfide bond formation (**Figure 6B**). We next examined previously published (Tsai et al., 2013) and more recent (Herz et al., 2024) transcriptome datasets derived from *E. multilocularis* primary cell cultures and various developmental stages. As shown in **Figure 6C**, *em-egf* expression was predominantly detected in protoscoleces and adult worms, with only minor expression in metacestodes. In contrast, *em-nrg* displayed stronger expression in the metacestode stage, and its transcripts were enriched following depletion of metacestode vesicles of stem cells, indicating preferential expression in post-mitotic cells (**Figure 6D**).

In summary, our analyses demonstrate that both identified *Echinococcus* EGF-like ligands exhibit structural hallmarks characteristic of this family of cytokine ligands, and that *em-nrg* represents a strong candidate for regulating stem cell dynamics in the metacestode stage.

### EmNRG interacts with EmER1 and EmER2

We next investigated whether the identified *Echinococcus* EGF-like ligands interact with the three parasite EGF receptors. For this purpose, we employed the MALAR yeast two-hybrid system, originally developed to detect extracellular interactions between peptide ligands and their cognate receptors (Li et al., 2016). Although this system was first designed for CXC-like chemokines and G-protein–coupled receptors (GPCRs), it has since been successfully adapted to study interactions between neuropeptides and GPCRs (Weth et al., 2020). Here, we applied it to examine potential interactions between EGF-like ligands and EGF receptors.

To generate ligand-bearing plasmids, we cloned cDNA regions encoding the complete extracellular domains of EmEGF, EmNRG, and human EGF, replacing the N-terminal signal peptide and transmembrane domains with vector-derived sequences. For receptor-bearing plasmids, we used the extracellular regions of EmER1, EmER2, EmER3, and the human EGFR HER1. Unfortunately, all ligand-bearing constructs for Em-EGF triggered yeast growth on quadruple dropout plates even with empty receptor vectors, rendering this system unsuitable for assessing EmEGF-receptor interactions. In contrast, ligand-bearing constructs for EmNRG showed only weak background growth in the absence of receptor baits.

**Figure 7:**
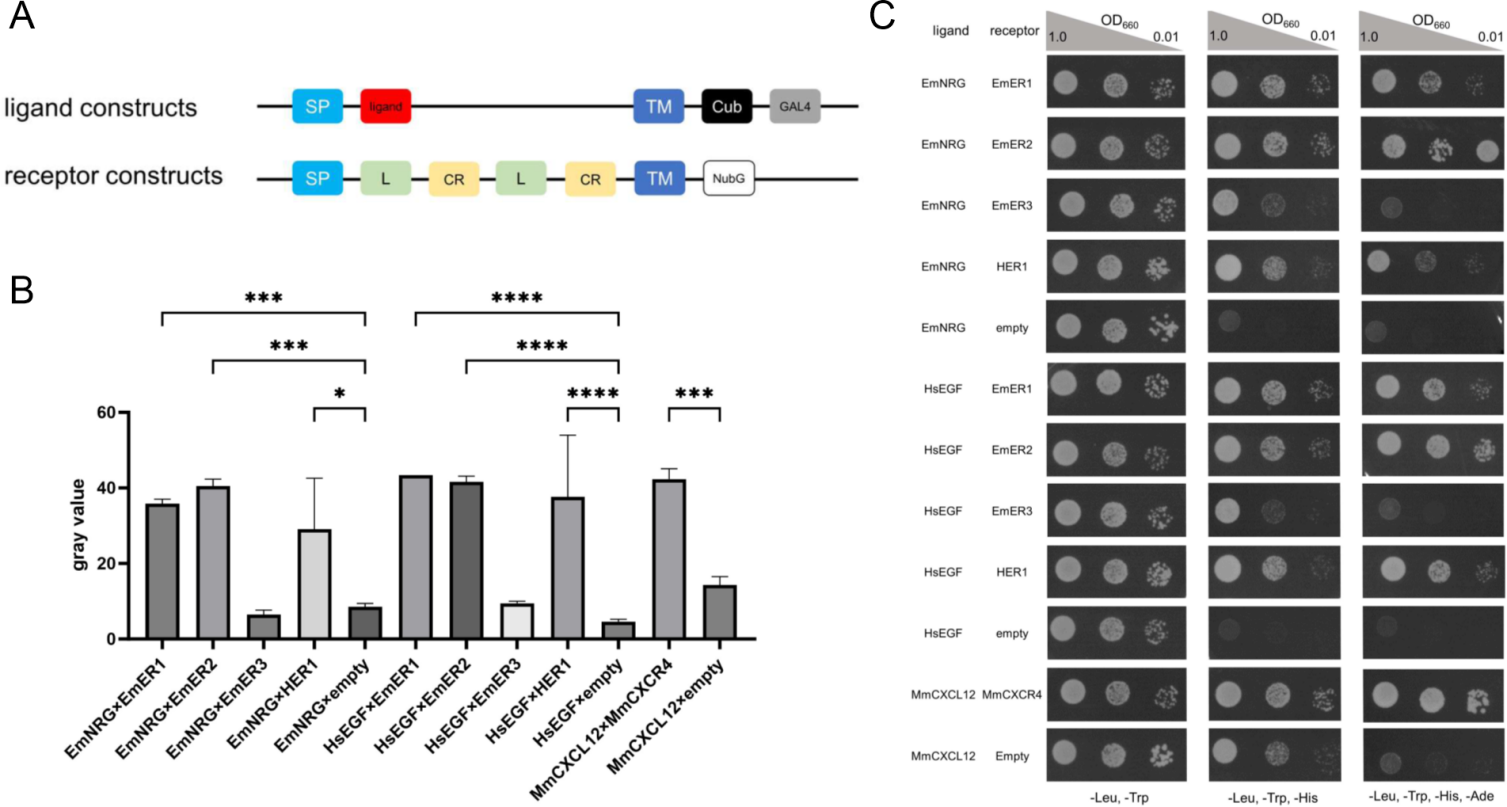
Interactions between *Echinococcus* EGF receptors and ligands. **(A)** Schematic image of ligand and receptor constructs for MALAR Y2H. SP: host cell signal peptide, L: receptor L domain, CR: cysteine-rich regions, TM: transmembrane region, NubG: N terminal half of ubiquitin with point mutation (Ile13Gly), Cub: C terminal half of ubiquitin. **(B)** Mean level of colony growth of different vector combinations (as indicated) quantified as gray value. Error bars represent standard deviation. One-way ANOVA followed by Tukey’s multiple comparison test was used to compare all plasmid combinations to one another, but only the comparisons of corresponding control are shown. P values less than 0.0332, 0.0002, and 0.0001 are indicated by *, ***, and **** respectively. MmCXCL12 and MmCXCR4 are murine chemokine ligands and receptors used as positive control. **(C)** Yeast growth on selective plates. Representative images of yeast transformants grown on double dropout (-Leu/-Trp, selective for plasmids), triple dropout (-Leu/-Trp/-His, indicating interactions under moderate stringency), and quadruple dropout plates (-Leu/-Trp/-His/-Ade, indicating interactions under high stringency). Inoculation densities are shown above, and plasmid combinations are shown to the left. Raw images were converted into grayscale and the background was subtracted with the algorism of sliding paraboloid.

Since our *Xenopus* oocyte expression experiments had already demonstrated that human EGF can activate EmER1 and, to a lesser extent, EmER2, we first tested these combinations. As shown in **Figure 7**, human EGF interacted with both parasite receptors in a manner comparable to its interaction with the cognate human receptor HER1. For EmER3, only very weak growth was detected with human EGF. Testing Em-NRG revealed robust interactions with EmER1 and EmER2, and even with human HER1 (**Figure 7**), but none with EmER3. Positive and negative controls included previously validated plasmids (Li et al., 2016) encoding the murine chemokine CXCL12 and its cognate receptor CXCR4, as well as CXCL12 with an empty vector. As expected, these controls showed either strong or negligible yeast growth, respectively.

Taken together, these findings indicate that Em-NRG functions as a ligand for EmER1 and EmER2 in *Echinococcus*.

### *em-nrg* is upregulated during germinative cell clonal expansion

**Figure 8:**
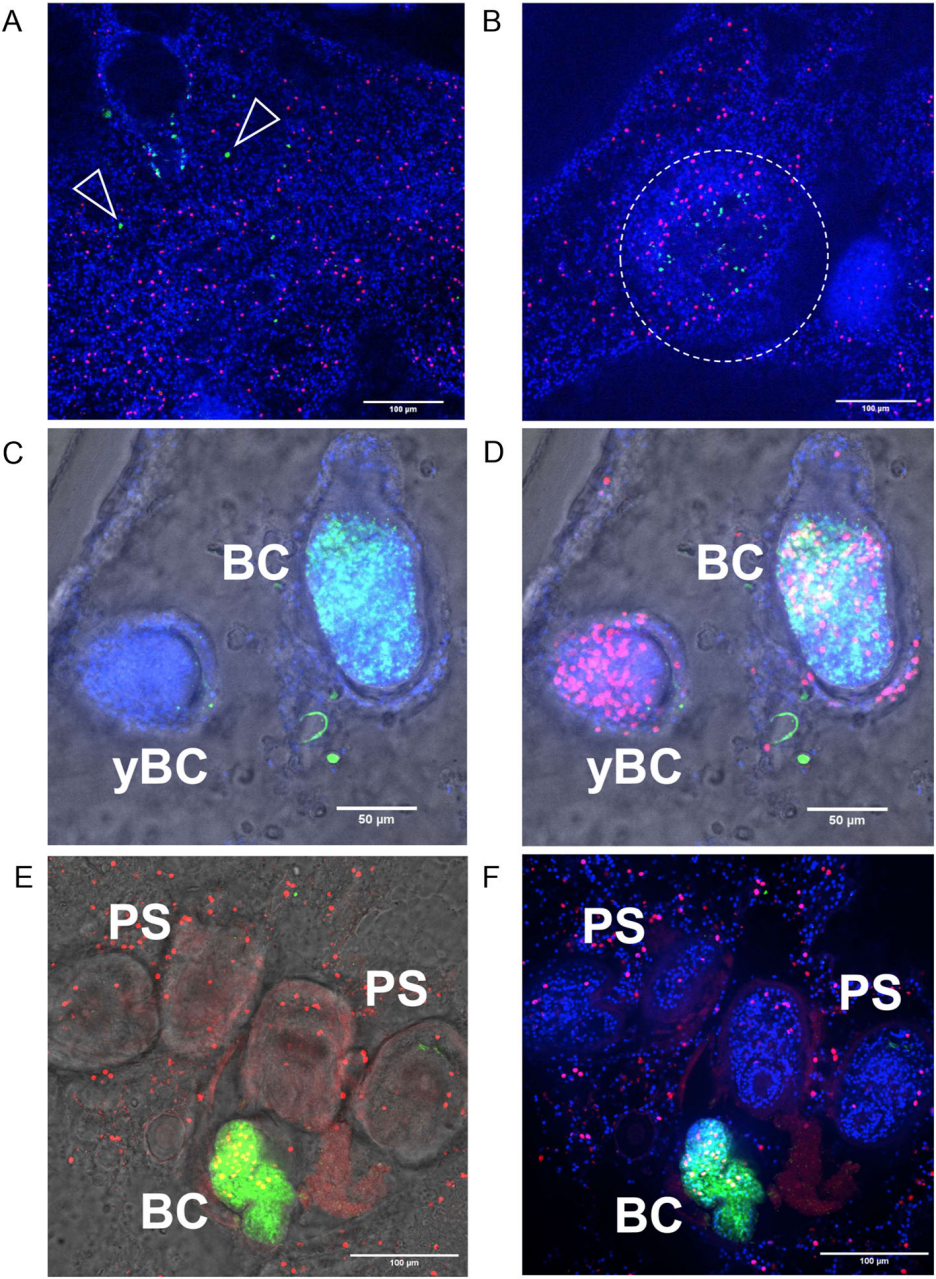
Expression of *em-nrg* in metacestode and brood capsules. Shown are WISH analyses for *em-nrg* expression in metacestode vesicles after 5 h EdU pulse. **(A)** Metacestode vesicle without brood capsules. Single, WISH+/EdU- cells are marked by open triangles. **(B)** Germinal layer with local concentration of WISH+ cells (dashed circle). **(C, D)** Vesicle with young (yBC) and advanced (BC) brood capsules. **(C)** shows green, blue channels and bright field. **(D)** shows merge of all channels and bright field. **(E, F)** Vesicle with advanced brood capsule (BC) and developed (dormant) protoscoleces (PS). **(E)** Shows green, red channels and brightfield, **(F)** shows merge of blue, green, and red channels. Channels are red (EdU, S-phase stem cells), green WISH (*em-nrg*), blue (DAPI, nuclei). Scale bars represent 100 µm in **(A, B, E, F)**, and 50 µm in **(C, D)**.

Under steady-state conditions, planarian stem cells are thought to divide asymmetrically, producing one self-renewing stem cell and one differentiating progeny (Wagner et al., 2012). To assess *em-nrg* expression under comparable conditions, we performed *em-nrg*–specific WISH on *in vitro* cultivated metacestode vesicles, combined with EdU incorporation to label S-phase germinative cells. As shown in **Figure 8A**, only very few (<1%) cells in the germinal layer stained positive for *em-nrg*, and none had incorporated EdU. Occasionally, we observed foci containing ten or more *em-nrg*+/EdU- cells (**Figure 8B**), but these were rare (2–3 per vesicle) and not present in every vesicle. Early brood capsules lacked *em-nrg* expressing cells, whereas strong signals were detected in more advanced brood capsules (**Figures 8C–F**). Toward the end of protoscolex formation, when germinative cell proliferation typically ceases (Koziol et al., 2014), no *em-nrg*+ cells were detected (**Figures 8E, F**). These observations indicate that under steady-state conditions *em-nrg* is expressed in a small subset of post-mitotic cells in the germinal layer, consistent with prior transcriptomic analyses of stem cell depleted metacestode vesicles (Herz et al., 2024). In those analyses, *em-nrg* expression increased significantly following stem cell depletion (Herz et al., 2024), further suggesting that the gene is primarily expressed in post-mitotic cells.

**Figure 9:**
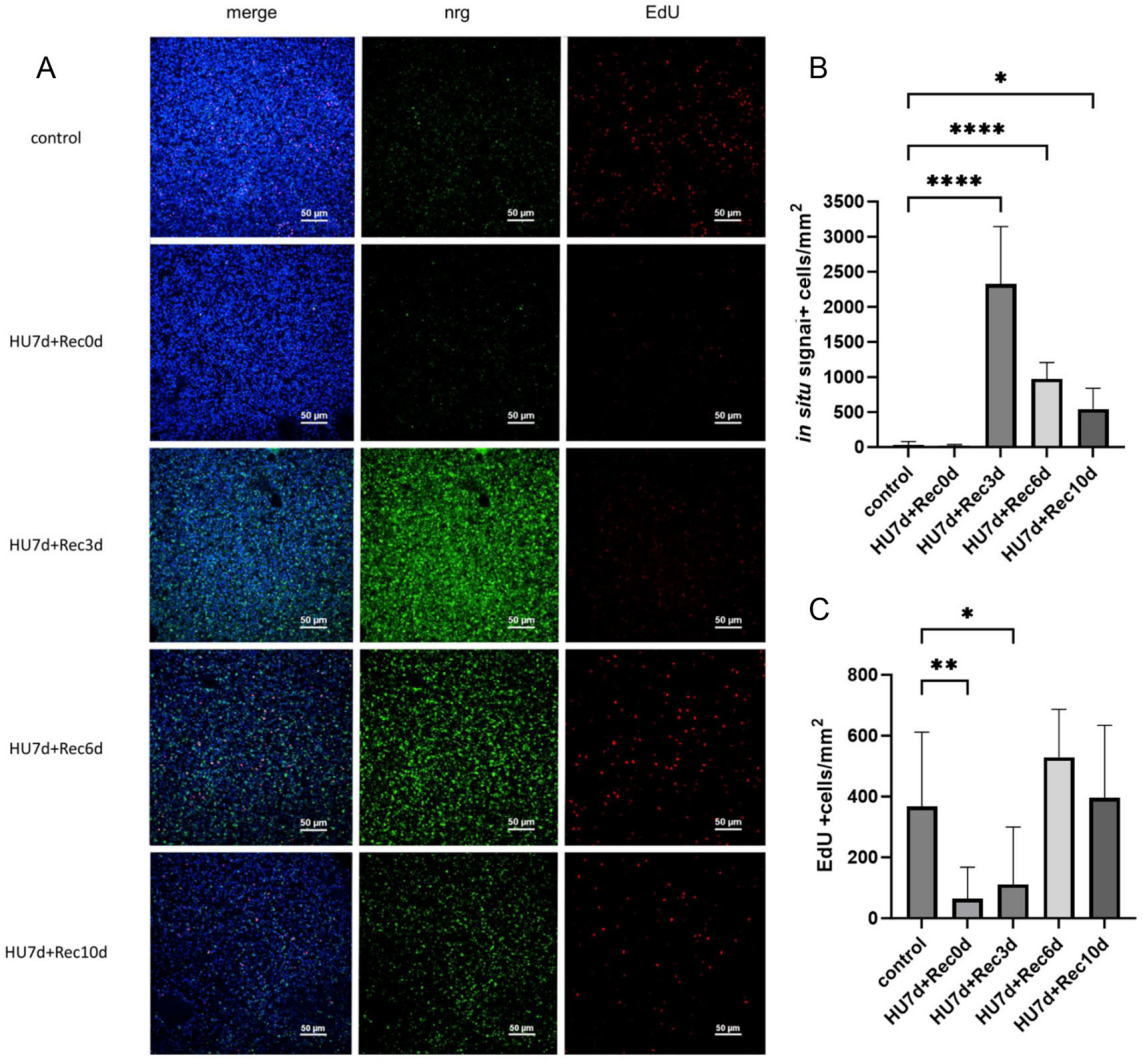
Expression of *em-nrg* during clonal germinal cell expansion. **(A)** Combined EdU incorporation/WISH assay. Displayed are single confocal slices of the germinal layer of metacestode vesicles after HU treatment and during clonal expansion of stem cells. ‘control’, vesicles without treatment; HU 7d, vesicle after HU treatment for 7 days; Rec, recovery for 3, 6, 10 days as indicated. Channels are green (WISH, em-nrg), red (EdU, S-phase germinative cells), blue (DAPI, nuclei). **(B)** Mean number of cells with WISH signal (per mm^2^). **(C)** Mean number of EdU+ cells (per mm^2^). Error bars represent standard deviation in both cases. One-way ANOVA followed by Dunnett’s multiple comparison tests was used to compare the control and each treatment condition. P values less than 0.0332, 0.0021, and 0.0001 are summarized with *, **, and **** respectively.

In metacestode vesicles, a 7 day hydroxyurea treatment markedly reduces the germinative cell population, after which the remaining cells undergo several rounds of clonal expansion to reconstitute the stem cell pool (Koziol et al., 2014). Under these conditions, germinative cells are expected to divide predominantly symmetrically. We therefore examined *em-nrg* expression after stem cell depletion. Vesicles were treated for 7 days until only few stem cells remained, then allowed to recover for 10 days. During recovery, we quantified EdU+ germinative cells and assessed *em-nrg* expression by WISH. As shown in **Figure 9**, immediately after depletion only few EdU+ cells were present and *em-nrg* expression remained relatively low. After 3 days of recovery, EdU+ cells increased modestly, consistent with previous reports that most metacestode stem cells cycle within ~3 days (Herz et al., 2024; Koziol et al., 2014), whereas *em-nrg*+ cells rose sharply from near zero to ~2,250 cells per mm² of metacestode tissue. After 6 days of recovery, EdU+ cells increased steeply to ~500 per mm², while *em-nrg*+ cells declined slightly (**Figure 9**). At 10 days, ~400 EdU+ cells per mm² were still observed, and *em-nrg*+ cells decreased further (**Figure 9**). Collectively, these data indicate that *em-nrg* is particularly highly expressed in metacestode tissue during stem-cell clonal expansion. Notably, even under these conditions, *em-nrg*+ cells were only rarely EdU+, again pointing to predominant expression in post-mitotic cells.

### *em-nrg* is required for metacestode development from stem cells

**Figure 10:**
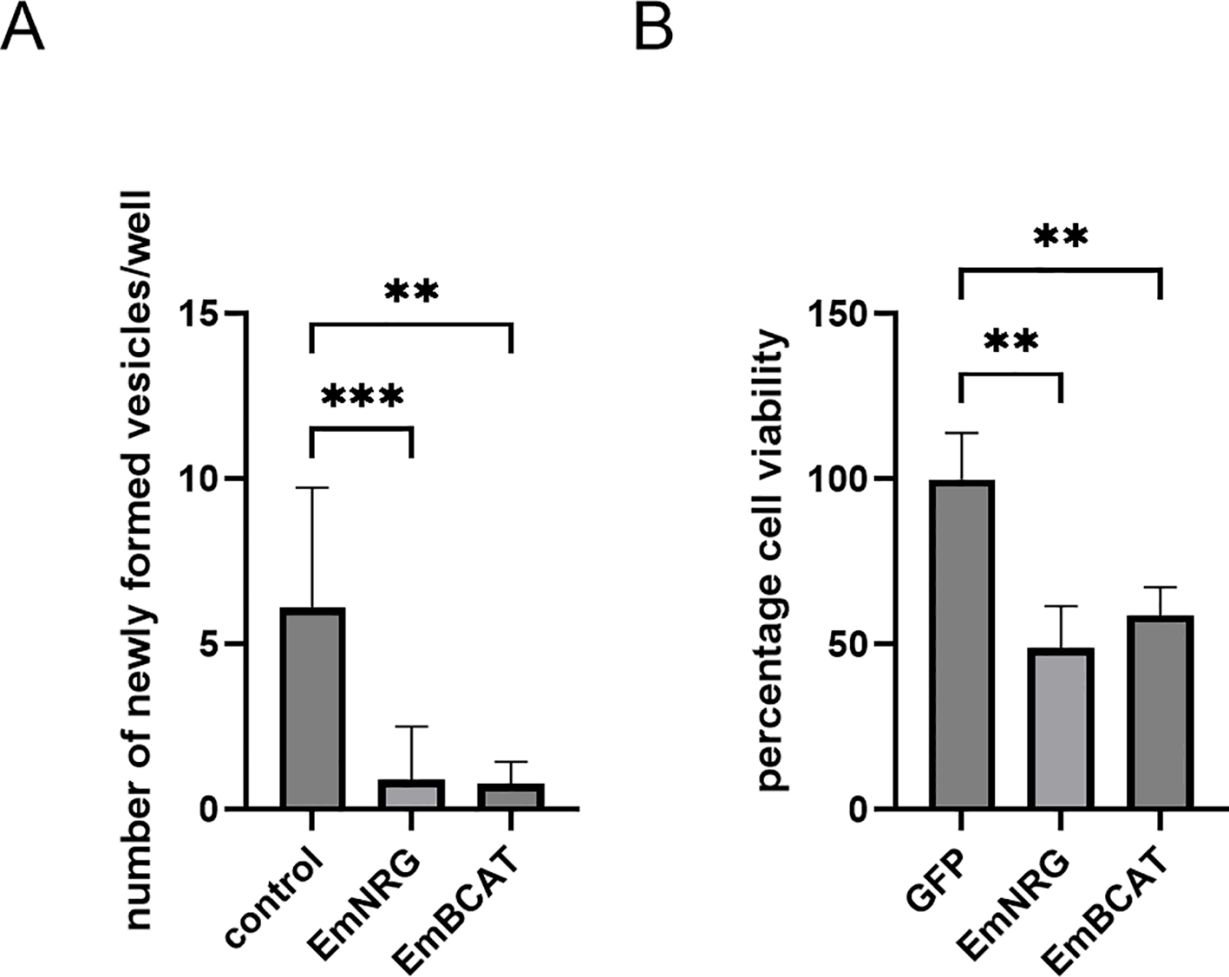
*em-nrg* is required for metacestode vesicle formation. Displayed are the results of siRNA-based RNAi knockdown of *em-nrg*. **(A)** Formation of mature metacestode vesicles from primary cell cultures after 21 days. The numbers of regenerated vesicles were analyzed by Kruscal-Wallis test followed by Dunn’s test. **(B)** Cell viability of primary cell cultures after 3 days. The normalized cell viability was analyzed by one-way ANOVA followed by Dunnet’s test. P values less than 0.0021, and 0.0002 are summarized with **, and ***, respectively. EmBCAT indicates results for the β-catenin encoding *E multilocularis* gene *bcat-1* (Herrmann et al., 2025), which was used as a positive control.

We next investigated whether RNAi-mediated knockdown of *em-nrg* in *E. multilocularis* primary cell cultures affects regeneration into metacestode vesicles. To this end, we applied a previously established RNAi protocol for *Echinococcus* primary cells (Spiliotis et al., 2010). As shown in **Supplementary Figure S4**, *em-nrg* transcript levels were reduced to approximately 70% compared with GFP and random controls. Notably, both metacestode vesicle formation and the viability of primary cell cultures were significantly impaired following *em-nrg* knockdown (**Figure 10**), indicating that expression of this EGF-like ligand gene is essential for proper stem cell function and parasite development.

### *emer1* is expressed in stem cell progeny

Previous analyses by Lei et al. (2016) showed that the majority (~85%) of *Smed-egfr3^+^* cells in *Schmidtea mediterranea* are neoblasts (i.e. also positive for the stem cell marker *smedwi1*), consistent with the role of the encoded receptor in regulating symmetric versus asymmetric neoblast divisions. In *Echinococcus*, however, our own data did not reveal a significant decrease in *emer1* transcript abundance in metacestode vesicles following stem cell depletion by hydroxyurea treatment (Herz et al., 2024). Moreover, as shown above, only ~9% of *emer1^+^* cells co-stained with EdU after a 5 h EdU pulse, marking S-phase stem cells. To determine whether *emer1* might be expressed in stem cell progeny or in late phases of the germinative cell cycle, we performed pulse-chase experiments consisting of a 5 h EdU pulse followed by *emer1*-specific WISH after 72 h. This timing was chosen because previous work (Herz et al., 2024; Koziol et al., 2014; Cheng et al., 2017) indicated that most germinative cells complete a division cycle within approximately three days.

**Figure 11:**
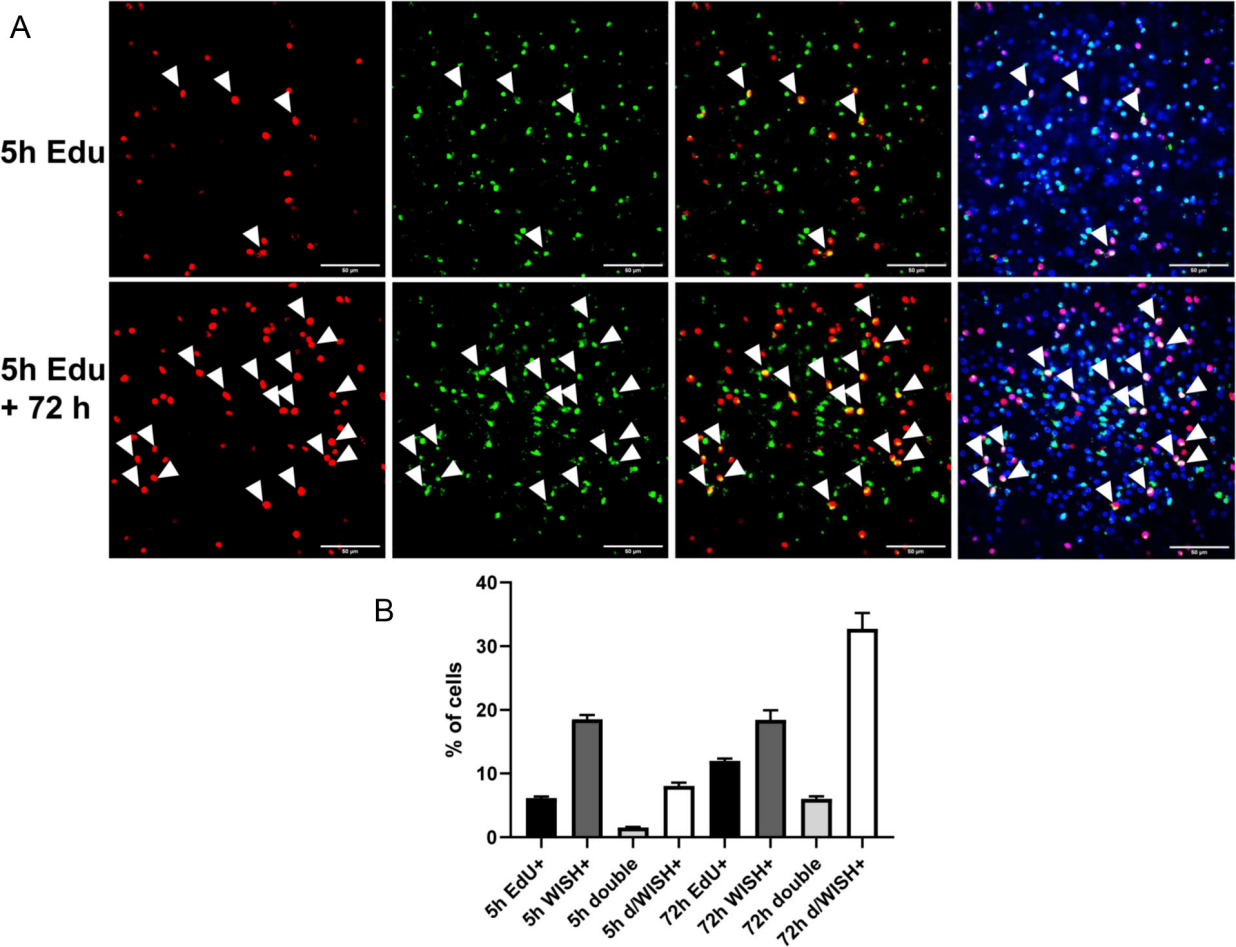
*emer1* is expressed in germinative cell progeny. **(A)** WISH/EdU analyses on metacestode vesicles after a 5 h EdU pulse (upper panel) and after 5 H EdU pulse plus 72 h recovery phase (lower panel). Shown are single confocal slices of, from left to right, red channel, green channel, red+green channel, merge of all three channels. Channels were red (EdU, S-phase stem cells), green (WISH, *emer1*), blue (DAPI, nuclei). Cells double positive for EdU and WISH are marked by white triangles. Size bar represents 50 µm in all cases. **(B)** Cell counts (% of all cells) of EdU+, WISH+, and WISH+/EdU+ (double) cells after 5 h EdU pulse (5h) and 5 h EdU plus 72 h recovery (72 h). 5h d/WISH+ indicates the relation of double positive (WISH+/EdU+) cells to WISH+ cells. Error bars indicate standard deviation.

As shown in **Figure 11**, immediately after the 5 h EdU pulse followed by WISH, we detected 6.2 ± 0.2% (n = 8244) EdU^+^ cells, 18.4 ± 0.6% *emer1^+^* cells, and 1.5 ± 0.1% double-positive cells. This confirms our above results and indicates that roughly 8.2 ± 0.5% of all *emer1^+^* cells correspond to S-phase stem cells. After a 72 h chase, we counted 11.9 ± 0.3% (n = 8086) EdU^+^ cells, indicating that most labeled stem cells had undergone mitosis. At this time point, 18.1 ± 1.3% of all cells were *emer1^+^*, with 6.1 ± 0.3% positive for both signals. These findings suggest that approximately one third (32.6 ± 2%) of all *emer1^+^* cells represent stem cell progeny or germinative cells in the terminal phase of the cell cycle.

## Discussion

Totipotent germinative cells are the key drivers of growth and proliferation in the *Echinococcus multilocularis* metacestode within its intermediate host. Representing about 20–25% of all metacestode cells, they constitute the only mitotically active cell population (Koziol et al., 2014; Brehm and Koziol, 2017). Under conditions of normal tissue turnover, these cells likely divide asymmetrically to yield one self-renewing stem cell and one differentiating daughter cell (Koziol et al., 2014; Herz et al., 2024). When stem cell numbers drop drastically, the remaining germinative cells proliferate clonally, apparently via symmetric divisions. The molecular mechanisms controlling the balance between asymmetric and symmetric division must therefore be tightly regulated. By integrating receptor characterization, functional interference, pharmacological inhibition, and ligand identification, our study now provides a first framework for understanding how EGF signalling might regulate stem cell dynamics in *E. multilocularis*.

Studies in free-living planarians have shown that EGF signaling regulates asymmetric division of neoblast stem cells (Lei et al., 2016). Consistent with this, EGF signaling has also been implicated in *Echinococcus* stem cell biology: exogenous human EGF stimulates metacestode stem cell proliferation and activates the previously characterized receptor EmER1 (Spiliotis et al., 2003; Cheng et al., 2017). Building on these findings, our present data indicate that *Echinococcus* stem cell division is at least partly controlled through interactions between the newly characterized EGF-like ligand EmNRG and its cognate receptor EmER1.

Our structural analyses identified EmNRG as a member of the neuregulin subfamily of EGF-like molecules, known to regulate stem cell division in vertebrates and planarians (Marchionni, 2014). Using the MALAR yeast two-hybrid system, we detected robust and specific interactions between EmNRG and EmER1 as well as EmER2, whereas EmNRG did not interact with EmER3 or empty vectors. The system’s internal controls, interactions between human EGF and EmER1 or human HER1, validated the assay’s specificity. These results identify EmNRG and EmER1 as a physiologically relevant receptor–ligand pair in *Echinococcus* EGF signaling.

Expression and functional data suggest that this interaction influences the decision between symmetric and asymmetric stem cell division. Both *emer1* and *em-nrg* are predominantly expressed in either stem cell progeny or post-mitotic cells, respectively, which are stages critical for fate determination. We therefore propose a model in which post-mitotic cells within the stem cell niche respond to local germinative cell abundance. When stem cells are underrepresented, *em-nrg* expression increases to promote clonal expansion. This interpretation is supported by the strong *em-nrg* up-regulation observed in stem-cell-depleted metacestode vesicles, during brood capsule formation, and in localized germinal layer regions exhibiting elevated *em-nrg* activity. Such regulation likely ensures a sufficient stem cell pool for protoscolex formation or compensates for transient local depletion. It is also possible that local stem cell depletion in *Echinococcus* induces signals similar to those observed in *H. miamia* that led to neuregulin induction after injury (Stevens et al., 2025) since both are expected to affect local stem cell pools. We further suggest that *emer1* acts toward the end of the germinative cell cycle or in post-mitotic progeny, directing cell fate toward self-renewal in the presence of EmNRG and toward alternative lineages in its absence.

Important open questions remain. It is still unclear whether *Echinococcus* expresses additional EmER1 ligands (possibly EmEGF) promoting differentiation and whether fate decisions occur before mitosis, through asymmetric receptor distribution, or after cell division via cell-cell communication. Similar issues have been addressed in planarians using specific antibodies against EGF receptors and ligands (Lei et al., 2016). Developing comparable tools for *Echinococcus* will be essential to elucidate the mechanisms underlying EmER1/EmNRG-mediated regulation of symmetric and asymmetric division. Such efforts are currently underway in our laboratory.

From a therapeutic perspective, both our RNAi and inhibitor assays identify EmER1 as the most probable *in vivo* target of afatinib. In the *Xenopus* expression system, EmER1 activity was clearly inhibited by afatinib at concentrations ≥ 100 nM. Consistently, RNAi-mediated *emer1* knockdown markedly impaired vesicle formation from primary cell cultures, whereas knockdown of *emer2* or *emer3* showed no effect. The strong *in vitro* and *in vivo* anti-parasitic efficacy of afatinib reported earlier (Cheng et al., 2017) therefore most likely results from EmER1 inhibition.

Interestingly, the amino acid residue critical for afatinib binding in mammalian EGFRs (Cys797) is replaced by serine in EmER1. Afatinib, an aniline-quinazoline–based second-generation EGFR inhibitor, covalently binds to this reactive cysteine within the ATP-binding pocket of human EGFR, thereby suppressing kinase activity and receptor dimerization (Nelson et al., 2013). Substitution of Cys797 by serine is known to confer partial resistance to third-generation EGFR inhibitors in human cancers (Grabe et al., 2018). Among the three *Echinococcus* EGFR homologs, only EmER3 retains the cysteine at this position; thus, afatinib may also inhibit EmER3, potentially even more efficiently than EmER1. However, since *emer3* knockdown did not impair vesicle formation, the observed anti-parasitic effects are best explained by EmER1 inhibition.

The structural divergence between EmER1 and human EGFRs in the inhibitor-binding pocket offers an opportunity to design parasite-selective inhibitors. A promising route would combine AI-based *in silico* screening of large compound libraries—an approach we recently applied successfully to *Echinococcus* PIM kinase (Koike et al., 2022)—with biochemical validation using recombinant EmER1 in the *Xenopus* expression system. AI-assisted virtual screening relies on differential binding-affinity datasets for multiple compounds (Kim et al., 2020). Hence, the comparative data of the inhibition against EmER1 and of the anti-parasite efficacy are valuable for rational drug design. In addition, the data of osimertinib deserves attention because it showed stronger effects against EmER1 in Xenopus expression system than against HER1.

While this study provides new insights into EGF signalling in *E. multilocularis*, several limitations should be acknowledged. First, technical constraints limited direct biochemical analyses of ligand–receptor interactions for all candidate ligands. Second, the lack of cell-type-specific genetic tools currently restricts precise dissection of signalling dynamics at the single-cell level. Finally, although our data strongly support a role for EmER1 and EmNRG in regulating stem cell behavior, the downstream signalling mechanisms remain to be fully elucidated. Future studies combining advanced imaging, improved functional genomics approaches, and parasite-selective inhibitor design will be essential to address these questions.

## Conclusions

We have identified the receptor tyrosine kinase EmER1 as a key molecular target of afatinib, which exhibits potent anti-parasitic activity both *in vitro* and *in vivo*. Together with data on structurally related inhibitors such as osimertinib and dacomitinib, these results provide a solid foundation for the rational development of novel therapeutics directed against EmER1—potentially through AI-guided compound screening and validation in the *Xenopus* system. Furthermore, we discovered a parasite-encoded ligand, EmNRG, that is strongly upregulated during clonal expansion of germinative cells and required for metacestode vesicle formation. While host-derived EGF, induced during hepatic regeneration, may modulate parasite proliferation, we propose that EmNRG functions as the principal ligand of EmER1 and that this receptor–ligand pair regulates the balance between asymmetric and symmetric divisions of germinative cells. These insights advance our understanding of stem-cell regulation in *Echinococcus* and open new avenues for therapeutic intervention in alveolar echinococcosis. Importantly, these findings establish a conceptual and experimental framework for future mechanistic and translational studies targeting parasite stem cell regulation.

## Data Availability

The datasets presented in this study can be found in online repositories. The names of the repository/repositories and accession number(s) can be found in the article/**Supplementary Material**.
