# Supplementary material for: A neuregulin-like ligand and EGF receptor underpin Echinococcus multilocularis development
Source: Front Cell Infect Microbiol. 2026 Feb 20;16:1742233. doi: 10.3389/fcimb.2026.1742233 (PMC12963305; doi:10.3389/fcimb.2026.1742233)
Supplement: Supplementary file 2 [file DataSheet1.pdf]

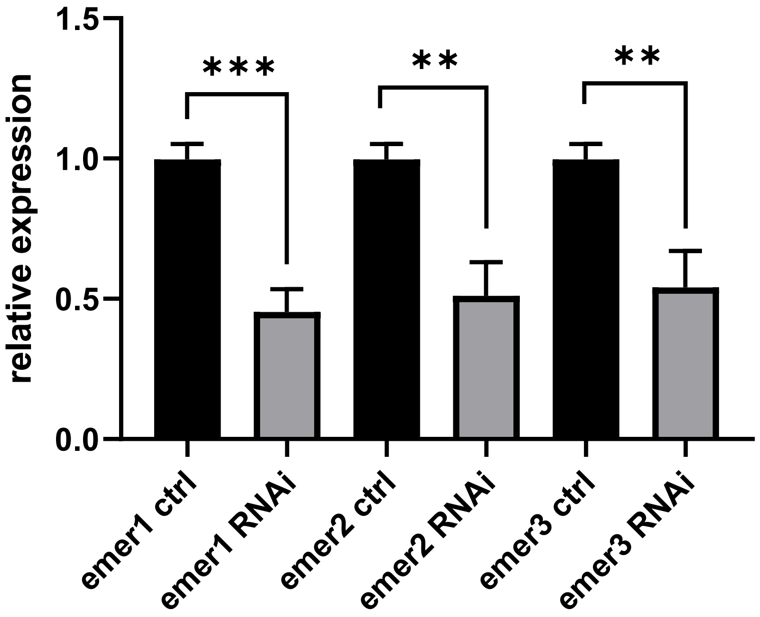

Figure S1. qRT- PCR analysis of *E. multilocularis* EGF receptor gene expression after RNAi. Expression of *emer1*, *emer2*, and *emer3* was analysed by qRT-PCR in cell cultures after RNAi and 3 days of incubation *in vitro*. Shown is relative expression in comparison to control gene *elp* (EmuJ_000485800). Indicated are relative expression values in control cultures (ctrl) and RNAi cultures (RNAi). Error bars indicate SD of three biological replicates. Statistical analysis indicates unpaired t-test, p value 0.0007 ***, 0.005 **.
