# Supplementary material for: A neuregulin-like ligand and EGF receptor underpin Echinococcus multilocularis development
Source: Front Cell Infect Microbiol. 2026 Feb 20;16:1742233. doi: 10.3389/fcimb.2026.1742233 (PMC12963305; doi:10.3389/fcimb.2026.1742233)
Supplement: Supplementary file 3 [file DataSheet2.pdf]

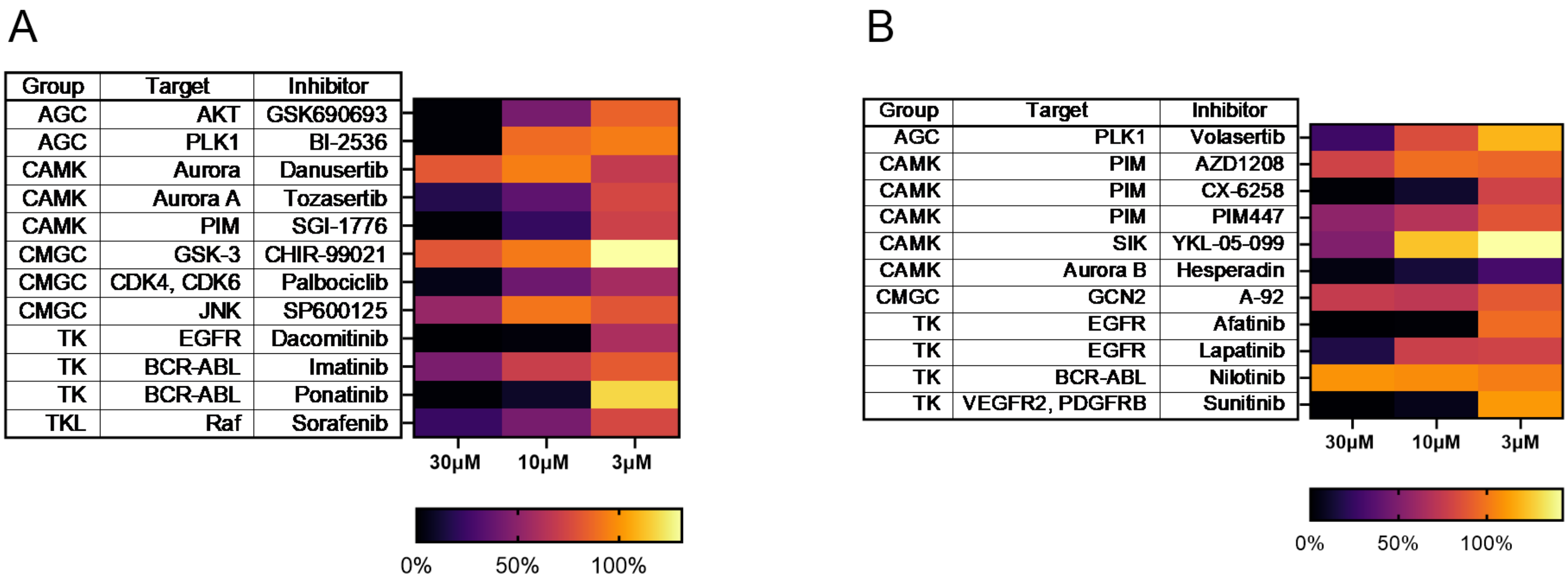

Figure S2. Activities of protein kinase inhibitors against E. multilocularis primary cells. (A-B) Heatmaps showing the effects of kinase inhibitors on primary cells of *E. multilocularis*. Color code below indicates the percentage of luminescence signal (proportional to the number of viable cells), normalized to signals from DMSO controls, after 3 days of incubation with 3-30 μM of inhibitor. Inhibitor names, human target proteins, and kinase groups are indicated in table to the left. (A) is the result of the screening with the second inhibitor list and (B) is that with the third inhibitor list. The results with the first inhibitor list were previously published (Koike et al, 2022).
