# Supplementary material for: A neuregulin-like ligand and EGF receptor underpin Echinococcus multilocularis development
Source: Front Cell Infect Microbiol. 2026 Feb 20;16:1742233. doi: 10.3389/fcimb.2026.1742233 (PMC12963305; doi:10.3389/fcimb.2026.1742233)
Supplement: Supplementary file 4 [file DataSheet3.pdf]

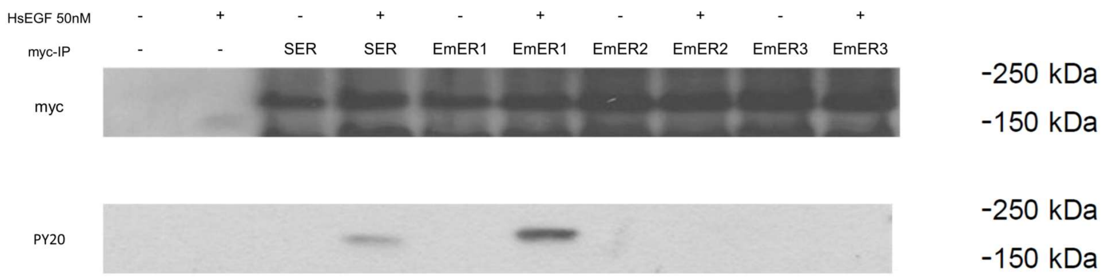

Figure S3 ; Phosphorylation of the recombinant protein 24 hours after the microinjection of cRNA or not (-), the oocytes were stimulated by human EGF for 15 hours. The recombinant protein expressed in the oocytes was purified with myc-IP from the lysates, and phosphorylation of the recombinant protein was evaluated by western blot with PY20 antibody.
