# Supplementary material for: A neuregulin-like ligand and EGF receptor underpin Echinococcus multilocularis development
Source: Front Cell Infect Microbiol. 2026 Feb 20;16:1742233. doi: 10.3389/fcimb.2026.1742233 (PMC12963305; doi:10.3389/fcimb.2026.1742233)
Supplement: Supplementary file 5 [file DataSheet4.pdf]

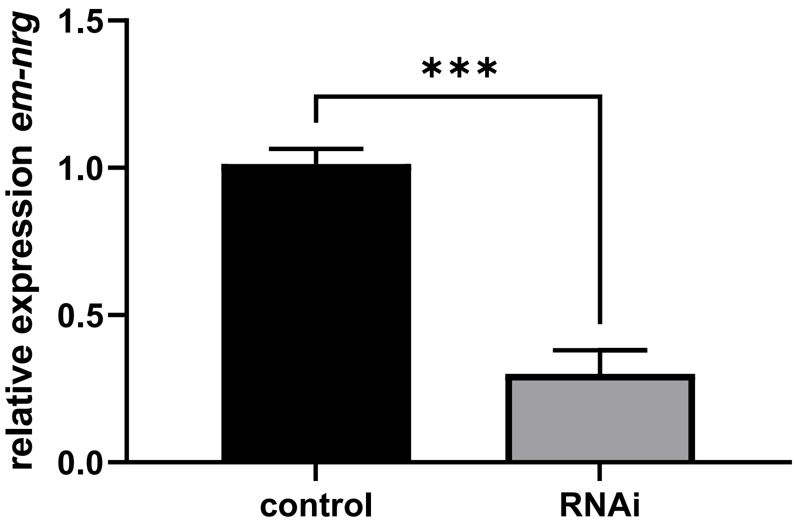

Figure S4. qRT- PCR analysis of *em-nrg* gene expression after RNAi. Expression of *em-nrg* was analysed by qRT-PCR in cell cultures after RNAi and 3 days of incubation in vitro. Shown is relative expression in comparison to control gene *elp* (EmuJ_000485800). Indicated are relative expression values in control cultures (control) and RNAi cultures (RNAi). Error bars indicate SD of three biological replicates. Statistical analysis indicates unpaired t-test, p value 0.0003 ***.
